# Probing novel epitopes on the *Plasmodium falciparum* circumsporozoite protein for vaccine development

**DOI:** 10.1038/s41541-024-01006-8

**Published:** 2024-11-18

**Authors:** Pascal S. Krenger, Magali Roques, Anne-Cathrine S. Vogt, Alessandro Pardini, Dominik A. Rothen, Ina Balke, Sophie T. Schnider, Mona O. Mohsen, Volker T. Heussler, Andris Zeltins, Martin F. Bachmann

**Affiliations:** 1 Department for BioMedical Research (DBMR), University of Bern, Bern, Switzerland; 2 Department for Rheumatology and Immunology, University Hospital of Bern, Bern, Switzerland; 3 Graduate School for Cellular and Biomedical Sciences (GCB), University of Bern, Bern, Switzerland; 4 Institute of Cell Biology, University of Bern, Bern, Switzerland; 5 Latvian Biomedical Research and Study Centre, Riga, Latvia; 6 Saiba AG, Pfäffikon, Switzerland; 7 Nuffield Department of Medicine, Centre for Cellular and Molecular Physiology (CCMP), The Jenner Institute, University of Oxford, Oxford, UK

**Keywords:** Protein vaccines, Malaria

## Abstract

RTS,S and R21 are the only vaccines recommended by the WHO to protect children from *Plasmodium falciparum (Pf)* clinical malaria. Both vaccines target the *Pf* sporozoite surface protein circumsporozoite protein (CSP). Recent studies showed that human antibodies neutralize *Pf* sporozoites most efficiently when simultaneously binding to the *Pf*CSP NANP repeat and the NPDP junction domain. However, neither RTS,S nor R21 targets this junction domain. To test the potential of the NPDP junction domain and other sites of *Pf*CSP as innovative vaccine targets, we developed multiple vaccine candidates based on cucumber mosaic virus-like particles (CuMV_TT_-VLPs). These candidates vary in several aspects: the number of targeted NANP repeats, the presence or absence of the junction domain, the cleavage site, and up to three NVDP repeats within the target sequence. Immunogenicity and efficacy studies were conducted in BALB/c mice, utilizing chimeric *Plasmodium berghei (Pb)* sporozoites, in which the endogenous CSP has been replaced by *Pf*CSP (*Pb/Pf*CSP). We observed a positive association between the number of targeted NANP repeats and the induction of specific IgM/IgG antibodies. Elevated humoral responses led to enhanced protection against parasitemia after *Pb/Pf*CSP sporozoite challenge. Especially high-avidity/affinity antibody formation and vaccine protection were NANP repeat-dependent. Intriguingly, vaccine efficacy was not enhanced by targeting sites on *Pf*CSP other than the NANP repeats. Our data emphasize the dominant role of the NANP repeat region for induction of protective antibodies. Furthermore, we present here novel malaria vaccine candidates with an excellent immunogenic profile that confer sterile protection in mice, even in absence of adjuvants.

## Introduction

Causing 249 million diseased individuals and 608 thousand death worldwide in 2022 malaria remains a serious health issue with a large socioeconomic impact^1^. Children under the age of 5, pregnant women, travelers from non-endemic regions, and immune-compromised people are at high risk of developing severe clinical symptoms that potentially lead to death^2^. Approximately 97% of malaria cases can be attributed to infection with the protozoan parasite *Plasmodium falciparum* (*Pf*), which is transmitted via the bite of female *Anopheles* spp. mosquitoes^3,4^. *Pf* is not only the most prevalent but also the deadliest of all *Plasmodium* species causing malaria^2,3^. Antimalarial drugs, vector control, and public health interventions have led to a worldwide decline in malaria cases between 2000 and 2015^5,6^. However, since then this trend has reversed due to rising parasite and mosquito drug resistance, necessitating the development of novel malaria preventive and treatment strategies^3^.

Epidemiological studies conducted in endemic areas beginning of the 20th century reported for the first time an age-related decrease in susceptibility to clinical malaria, suggesting the acquisition of adaptive immunity^7^. A passive transfer of IgG antibodies from immune adults to children who had malaria, performed in the 1960s, successfully reduced the parasitemia and clinical symptoms of the latter^8,9^. These observations identified the humoral immune response as a key player in malaria disease control and for the first time raised the hope for the development of a malaria vaccine. 60 years later RTS,S in combination with the liposome-based adjuvant AS01 that includes the immunostimulants MPL and QS-21 (Mosquirix™) as well as R21 formulated in Matrix-M are the only vaccine candidates that have achieved regulatory approval and that have been recommended by the WHO for *Pf* malaria prevention in children living in endemic regions^3,10,11^. RTS,S and R21 are both protein subunit vaccines based on the hepatitis B virus surface antigen (HBsAg) that target the circumsporozoite protein (CSP), one of the most abundant proteins of the *Pf* sporozoite surface^12,13^.

Once injected into the host skin during a mosquito blood meal, motile sporozoites enter the bloodstream and reach hepatocytes in the liver within hours post infection^2,4^. Binding of *Pf*CSP to the highly sulfated proteoglycans on hepatocytes leads to the processing of its N-terminus thus allowing hepatocyte invasion and thereby a first round of parasite replication within the host liver^14^. *Pf*CSP comprises a heparin sulfate binding site on the N-terminus followed by a central region consisting of up to 49 NANP repeats that are interspersed with NVDP motifs and a thrombospondin-like type I repeat (TSR) domain on the C terminus. A glycosylphosphatidylinositol (GPI)-modification anchors the *Pf*CSP in the sporozoite membrane^15–18^. The RTS,S vaccine targets amino acids 207–395 of the *Pf*CSP (NF54 strain) thereby including 19 of the immunodominant NANP repeats as well as known CD4^+^ T cell epitopes^19,20^. R21 targets the virtually identical site on *Pf*CSP but differs from RTS,S in terms of antigen density on the particle surface, as it is composed of a single *Pf*CSP-HBsAg fusion protein and is not expressed as mosaic particles^13^. Increased epitope density is a feature known to enhance immunogenicity^21,22^.

A large phase III trial conducted in 5–17-month-old children living in malaria-endemic regions in Africa revealed a vaccine efficacy of RTS,S against clinical malaria of 74% shortly after the third and last immunization which dropped to 28% 1 year later. In 6–12 weeks old infants of the same study, a vaccine efficacy of 63% was reported directly after immunization that waned to 11% after 1 year^23–25^. Analysis of anti-*Pf*CSP antibody titers in this cohort showed a rapid decrease in vaccine-induced serum antibodies. Only 30% of vaccine-induced antibodies were detectable 5 years post-immunization. Decreasing anti-*Pf*CSP antibody titers correlated with declined clinical efficacy, highlighting the important role of anti-*Pf*CSP antibodies for protection^26^. In adults, the data are limited to two smaller studies in which either no, or low (34%) and short-lived vaccine efficacy of RTS,S was found^27,28^. For R21 a phase IIb clinical trial performed in 5–17-month-old children living in Burkina Faso reported a 1-year efficacy of 77%^29^. However, data from long-term studies for R21 are still pending and have to prove if vaccine efficacy remains that high over time.

*Pf*CSP-specific antibody-mediated protection from malaria is based on several mechanisms. This includes interference with the sporozoite motility in the skin and blood circulation, inhibition of sporozoite transmigration through hepatocytes, and inhibition of hepatocyte infection^30–36^. Likewise, *Pf*CSP-specific antibodies activate Fc-mediated effector functions such as opsonization for phagocytosis, complement system, or NK cell-mediated cytotoxicity^37–40^.

Recent studies have demonstrated that monoclonal human antibodies neutralize *Pf* sporozoites most effectively when they simultaneously recognize two distinct epitopes of the *Pf*CSP: the NANP repeats and the conserved NPDP junction domain located between the internal cleavage site and the central repeat region^30,36,41,42^. The human monoclonal antibody CIS43 specifically binds to the junction domain and the NANP repeats conferring sterile protection in two mouse models of *Plasmodium* infection, underlining the importance of the junction domain for antibody-based *Pf* neutralization^41,43^. Interestingly, antibodies exclusively targeting the junction domain exhibited reduced effectiveness in neutralizing *Pf* sporozoites compared to antibodies that simultaneously bind to NANP repeats and the junction domain^30^. This reduced effectiveness could potentially be attributed to a diminishing antibody binding affinity toward the N-terminus. For example, the cleavage site-specific antibody 5D5 showed limited binding capacity for *Pf* sporozoites resulting in a low neutralization capacity^44^.

Both, the RTS,S and R21 vaccines are designed to specifically target the NANP repeat region, omitting the junction domain^19,20^. Also, the NVDP minor repeats, which have been identified as neutralizing epitopes, are missing in the design of both licensed malaria vaccines^45^. This indicates that the incorporation of the junctional domain or the NVDP repeats into the vaccine design could potentially lead to the development of an enhanced malaria vaccine.

Here, we tested the hypothesis of whether an improved malaria vaccine can be obtained by targeting the *Pf*CSP junctional domain in combination with the NANP repeats. For this purpose, we used cucumber mosaic virus-like particles that are engineered with a universal T cell epitope derived from the tetanus toxin (CuMV_TT_-VLPs)^46^ and genetically fused different epitopes of *Pf*CSP including the junction domain onto the particles’ surface. VLPs have a favorable immunogenic profile that relies on their high similarity to real viruses and is based on the following main characteristics. First, VLPs possess a highly repetitive surface structure that facilitates B cell activation through B cell receptor (BCR) cross-linking and that efficiently engages natural antibodies and the complement system. In turn, complement bound particles can bind to CD21 on B cells thereby activating these cells by signaling through the co-stimulatory molecule CD19^47,48^. Additionally, complement bound particles are more frequently uptaken by antigen presenting cells (APCs) and thereafter presented to T cells and specifically delivered to follicular dendritic cells (FDCs) for germinal center formation by CD21 mediated binding to B cells^49^. Second, similar to real viruses, VLPs carry on their inside nucleic acids. This is not infectious genetic material but mRNA derived from the expression system incorporated into the particles during particle formation. Prokaryotic RNA derived from *Escherichia coli* (*E. coli*) acts as TLR 3 (recognizing double-stranded RNA) and 7/8 (recognizing single-stranded RNA) ligand^22,50^. TLR stimulation induced by VLP-carried RNA accelerates germinal center development and promotes the formation of antigen-specific high-affinity IgG antibodies, including the IgG2a and IgG2c subclasses, as demonstrated for Qb-derived vaccines against influenza and cat allergy^51–53^. In particular, high-affinity/avidity IgG, as well as IgG2a/c subclass antibodies, were shown to strongly contribute to vaccine-mediated protection^22,52^. Third, the size of the VLPs allows free drainage into the lymphatic vessels so that they reach the B cells in native form. Importantly, the immunogenic character of VLPs is not exclusively to the particles but also applies to antigens displayed on the surface of these particles^21^. Thus, CuMV_TT_-VLP-based vaccines were proven to be highly immunogenic and effective in preclinical and clinical studies performed for SARS-CoV-2, peanut, and cat allergy^54–59^.

*Pf* sporozoites neutralizing antibodies were shown to be most efficient when simultaneously binding to the NANP repeat and the NPDP junction domain^13,14^. Since neither RTS,S nor R21 targets the junction domain, we tested if inclusion of the junction domain may improve the efficacy of *Pf*CSP-specific vaccine candidates^19,20^. Furthermore, we evaluated the dependency of the vaccines’ immunogenicity and efficacy on the number of incorporated NANP repeats and investigated other domains such as the cleavage site or the NVDP repeat region as novel target sites for vaccination. For this purpose, we have successfully produced different malaria vaccine candidates on the basis of CuMV_TT_-VLPs and subjected them to immunogenicity and efficacy studies in BALB/c mice. To model *Pf* malaria in mice, we employed a chimeric line of the rodent parasite *Plasmodium berghei* (*Pb)*, wherein the native *Pb*CSP was replaced by its *Pf* homolog. *Pb* is commonly used in malaria research to study pre-erythrocytic stages of the parasite due to its safety for humans, ease of genetic manipulation, and its close resemblance to human malaria parasites^60^. Our data revealed a strong correlation between *Pf*CSP-specific humoral immune responses and the number of targeted NANP repeats. In contrast, other regions than the central repeats of *Pf*CSP seemed to play a subordinate role in the vaccines’ immunogenicity. Elevated IgM and IgG titers, particularly high-avidity/affinity IgG antibodies elicited by NANP repeats following vaccination, were associated with enhanced vaccine efficacy, as evidenced by achieving sterile protection against parasite infection.

## Results

### CuMV_TT_-based malaria vaccine candidates were effectively produced in *E. coli*

**Fig. 1:**
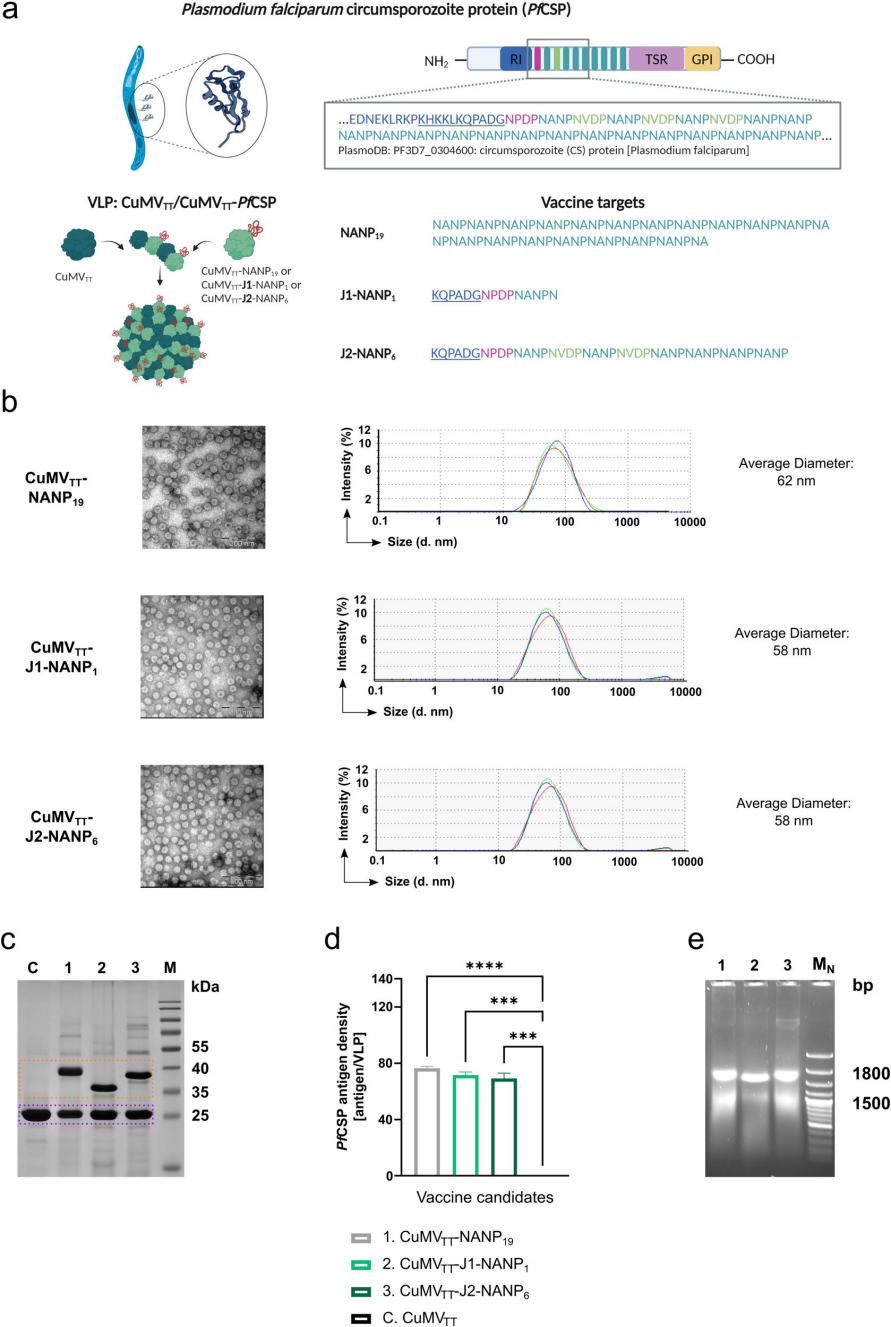
Design and characterization of CuMV_TT_-based malaria vaccine candidates targeting different sites of *Pf*CSP. **a** Schematic representation of the vaccine candidate designs. Vaccine candidates are based on CuMV_TT_-VLPs genetically fused to different amino acid sequences of *Pf*CSP (PlasmoDB: PF3D7_0304600). The sequences include 19 NANP repeats (CuMV_TT_-NANP_19_), KQPGDGNDPDNANPN (CuMV_TT_-J1-NANP_1_), and KQPGDGNDPDNANPNVDPNANPNVDPNANPNANPNANPNANP (CuMVTT-J2-NANP_6_). Particles are expressed as mosaic VLPs, comprising CuMV_TT_ subunits fused to parts of *Pf*CSP and subunits that remain unfused. The scheme was created with BioRender.com. **b** TEM and DLS of vaccine candidates after expression in *E. coli*, scale bar 200 nm. **c** 12% SDS–PAGE of C. CuMV_TT_, 1. CuMV_TT_-NANP_19_, 2. CuMV_TT_-J1-NANP_1_, 3. CuMV_TT_-J2-NANP_6_, and M. Protein Marker after Coomassie staining. Protein bands derived from the fusion product of CuMV_TT_ to different amino acid sequences of *Pf*CSP are circled in orange, and CuMV_TT_-derived protein bands are circled in violet. **d** Antigen density of *Pf*CSP-derived amino acid sequences on the surface of the particles: 1. CuMV_TT_-NANP_19_, 2. CuMV_TT_-J1-NANP_1_, 3. CuMV_TT_-J2-NANP_6_, and C. CuMV_TT_. Antigen density was determined based on FIJI image J software analysis of protein band intensities from SDS–PAGE depicted in Fig. 1c^22^. **e** Agarose gel analysis of 1. CuMV_TT_-NANP_19_, 2. CuMV_TT_-J1-NANP_1_, 3. CuMV_TT_-J2-NANP_6_, and M_N_. nucleic acid marker depicting the VLP encapsulated RNA derived from the expression host. Statistical analyses were performed as follows: data are presented as mean ± SEM. In (**d**), one-way ANOVA with Tukey correction was used for comparison. Significance levels are denoted as follows: *p* < 0.001 (***), *p* < 0.0001 (****). *N* = 2.

Based on the CuMV_TT_ vaccine platform we designed three distinct vaccine candidates targeting different sequences of *Pf* CSP (PlasmoDB: PF3D7_0304600). The first candidate, CuMV_TT_-NANP_19_, specifically targets 19 NANP repeats. The second candidate, CuMV_TT_-J1-NANP_1_, targets the amino acid sequence KQPGDGNDPDNANPN, thereby including part of the internal cleavage side, the junction region, and a single NANP repeat in an attempt to enhance induction of double-specific antibodies. CuMV_TT_-J2-NANP_6_, the third candidate, is an extended version of CuMV_TT_-J1-NANP_1_ additionally targeting two NVDP and five NANP repeats. All three vaccine candidates were expressed in *E. coli* as mosaic particles composed of CuMV_TT_ and CuMV_TT_ subunits that were genetically fused with *Pf*CSP-derived sequences (CuMV_TT_/CuMV_TT_-*Pf*CSP) similarly as previously described for the VLP Peanut vaccine (Fig. 1a)^58^. After expression, we performed ultracentrifugation for particle purification and endotoxin removal. Transmission electron microscopy (TEM) confirmed the successful formation of VLPs, displaying the characteristic icosahedral geometry and *T* = 3 symmetry typical of CuMV_TT_-derived VLPs for all vaccine candidates (Fig. 1b left)^46,58,61^. CuMV_TT_-J1-NANP_1_ and CuMV_TT_-J2-NANP_6_ particles exhibited an average diameter of 58 nm, as measured by dynamic light scattering (DLS), whereas CuMV_TT_-NANP_19_ particles displayed a slightly larger average diameter of 62 nm (Fig. 1b right). This difference in size can be attributed to the larger genetic sequence fused to CuMV_TT_-NANP_19_ particles compared to CuMV_TT_-J1/J2-NANP_1/6_ particles. For all vaccine candidates, DLS analysis revealed a single peak, indicating a uniform size distribution of the VLPs and suggesting the absence of impurities or aggregates (Fig. 1b right). We analyzed the particle composition of the three vaccine candidates using SDS–PAGE. CuMV_TT_-derived protein bands, with a molecular mass of 28 kDa, are circled in violet on the gel, while protein bands originating from fused subunits ranged in molecular mass from 37 to 40 kDa, depending on the size of the inserted *Pf*CSP sequence, and are highlighted by the orange frame (Fig. 1c). As previously described we calculated the insertion rate of the fused subunits into the particles, also referred to as antigen density, based on the protein band signal intensities observed in SDS–PAGE and quantified by FIJI Image J software^22^. For this purpose, we divided the protein signal intensity of the fused CuMV_TT_ subunits by the protein signal intensity of the unfused CuMV_TT_ subunit of the same sample. This ratio was then corrected for the different molecular masses and multiplied by 180, the total number of subunits of CuMV-VLPs^46^. This analysis revealed a consistent incorporation rate of *Pf*CSP-carrying subunits into the VLPs for all three vaccine candidates (Fig. 1d). Additionally, we confirmed the encapsulation of prokaryotic RNA, which acts as TLR 3 and 7/8 ligand, by agarose gel electrophoresis for all the produced vaccine candidates (Fig. 1e)^22^. In summary, the three malaria vaccine candidates, CuMV_TT_-NANP_19_, CuMV_TT_-J1-NANP_1_, and CuMV_TT_-J2-NANP_6_, were successfully produced. Each candidate exhibited intact and properly folded VLPs that encapsulate prokaryotic RNA. The only difference among these candidates is in their target sequences, while their overall particle integrity remains consistent.

### The number of NANP repeats targeted by the vaccine candidates is directly linked with the induction rate of *Pf*CSP-specific antibodies and the subsequent inhibition of sporozoite progression to the blood stage

**Fig. 2:**
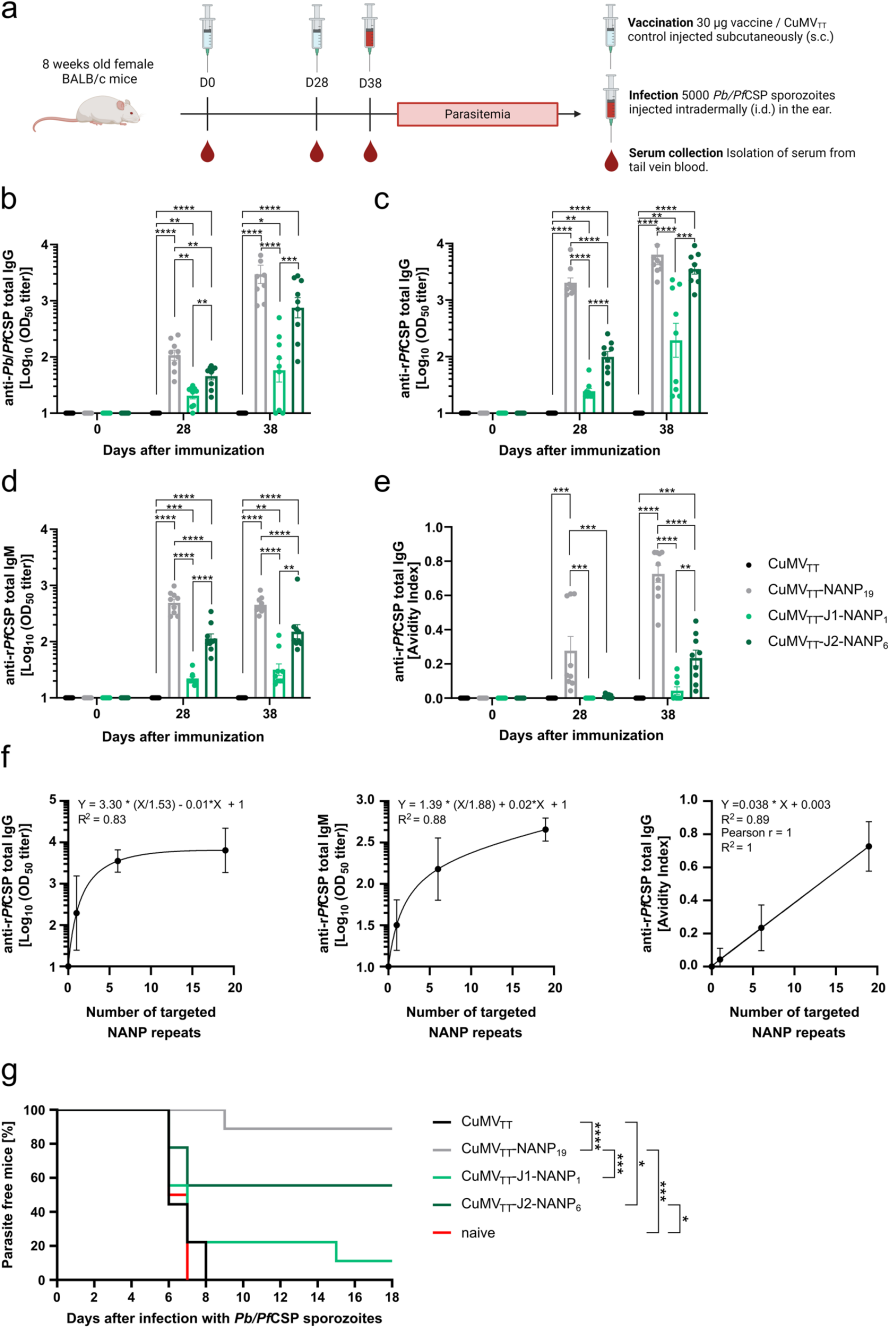
Immunogenicity and protective efficacy of CuMV_TT_-based malaria vaccine candidates. **a** Mouse model to evaluate the immunogenicity and protective efficacy of the different malaria vaccine candidates. 8-week-old female BALB/cOlaHsd mice were immunized on days 0 and 28 with 30 µg of the vaccine candidate, formulated in 100 µL PBS per mouse and administered s.c. On day 38, the mice were infected with 5000 *Pb/Pf*CSP sporozoites, delivered i.d. in the ear. Parasitemia was assessed daily by flow cytometry, checking for GFP-positive erythrocytes (*Pb/Pf*CSP infected). Blood samples were collected from the tail vein on days 0, 28, and 38, and serum was isolated for immunological analysis. The scheme was created with BioRender.com. **b** *Pb/Pf*CSP sporozoite-specific serum IgG measured by ELISA, Log_10_ OD_50_ is shown. **c** r*Pf*CSP-specific serum IgG measured by ELISA, Log_10_ OD_50_ is shown. **d** r*Pf*CSP-specific serum IgM measured by ELISA, Log_10_ OD_50_ is shown. **e** Avidity Index (proportion of high-avidity antibodies) of r*Pf*CSP-specific serum IgG measured by an adapted ELISA protocol using a 7 M urea washing step to remove low-avidity antibodies from the plate. **f** Association between vaccine-induced r*Pf*CSP-specific IgG or IgM titers and the number of NANP repeats targeted by the vaccine approximated using a saturation curve model (left and middle). Linear correlation between r*Pf*CSP-specific high-avidity IgG antibodies and the number of NANP repeats targeted by the vaccine (right). **g** Parasite-free mice after i.d. injection of 5000 *Pb/Pf*CSP sporozoites. Statistical analyses were performed as follows: data are presented as mean ± SEM. In (**b**–**e**), one-way ANOVA with Tukey correction was used for comparisons. In (**g**), statistical significance was assessed using the Log-rank (Mantel–Cox) test. Significance levels are denoted as follows: *p* < 0.05 (*), *p* < 0.01 (**), *p* < 0.001 (***), *p* < 0.0001 (****). *N* = 9. One representative of two comparable experiments is shown.

The immunogenicity and efficacy of the vaccine candidates were studied in a murine malaria model. For this purpose, we administered 30 µg of the vaccine candidate formulated in PBS via s.c. injection to 8-week-old female BALB/cOlaHsd mice on days 0 and 28. As control group, we included mice immunized with unmodified CuMV_TT_ particles. 10 days after the booster injection, on day 38, each mouse was infected by an intradermal (i.d.) injection of 5000 *Pb/Pf*CSP sporozoites. These *Pb* chimeric sporozoites have their endogenous CSP gene replaced by the *Pf* counterpart and constitutively express cytosolic GFP, allowing tracking by flow cytometry (see “Methods” section; Triller et al.^31^). The i.d. injection was chosen as it represents the natural route of *Plasmodium* infection, and the dermis is the first site for antibodies to fulfill their protective function^4,35,62^. Serum samples were collected on days 0 (immunization), 28 (boost), 38 (before sporozoite challenge) and analyzed by ELISA for total IgG specific for *Pb/Pf*CSP sporozoites. Additionally, IgG as well as IgM antibodies specifically binding to recombinantly expressed *Pf*CSP (r*Pf*CSP) were assessed (Fig. 2a and Supplementary Fig. 1).

Before immunization (D0) no *Pb/Pf*CSP-specific IgG was detected, nor were r*Pf*CSP-specific IgG or IgM antibodies present (Fig. 2b–d). 4 weeks following the initial immunization (D28), significantly higher *Pb/Pf*CSP-specific IgG titers were observed in mice vaccinated with CuMV_TT_-NANP_19_ compared to those vaccinated with junction domain-targeting candidates (Fig. 2b). Among the junction domain-targeting vaccine candidates, CuMV_TT_-J2-NANP_6_ induced the most robust antibody response (Fig. 2b). The booster injection further elevated *Pb/Pf*CSP-specific IgG antibody titers across all groups (Fig. 2b). After boost, the highest levels of specific IgG antibodies were observed in mice immunized with CuMV_TT_-NANP_19_, followed by those immunized with CuMV_TT_-J2-NANP_6_ and CuMV_TT_-J1-NANP_1_ (Fig. 2b). The development of r*Pf*CSP-specific IgG and IgM antibodies mirrored this pattern, with IgM antibody levels remaining stable post boost (Fig. 2c, d). In general, titers measured for the r*Pf*CSP-specific antibodies were slightly higher than those for whole *Pb/Pf*CSP sporozoites. This phenomenon may be attributed to the fact that the *Pf*CSP protein on the surface of sporozoites is less accessible to antibodies compared to the recombinant protein. Alternatively, this might be a matter of the quantity of coated *Pf*CSP protein, which may have been higher when we coated the ELISA plates with r*Pf*CSP. No *Pb/Pf*CSP- or r*Pf*CSP-specific IgG/IgM antibodies were detected in mice immunized with CuMV_TT_ control particles (Fig. 2b–d).

We performed a modified ELISA, that includes an additional washing step with 7 M Urea to determine the induction of high-avidity IgG antibodies specific for r*Pf*CSP after vaccination^63^. On day 28 (pre-boost), ~30% (Avidity Index (AI): 0.3) of the IgG antibodies induced by the CuMV_TT_-NANP_19_ vaccine exhibited high avidity for the r*Pf*CSP antigen (Fig. 2e). This average was significantly influenced by three samples with an AI of around 0.6 (Fig. 2e). In contrast, no high-avidity r*Pf*CSP-specific IgG antibodies were detected in the sera of mice immunized with CuMV_TT_-J2-NANP_6_ or CuMV_TT_-J1-NANP_1_ on day 28 (Fig. 2e). Following the booster dose (D38), the average proportion of high-avidity r*Pf*CSP-specific IgG antibodies in the CuMV_TT_-NANP19 group increased to ~70% (Fig. 2e). Around 20% of high-avidity r*Pf*CSP-specific IgG antibodies were found in the sera of mice immunized with CuMV_TT_-J2-NANP_6_ on day 38 (Fig. 2e). The CuMV_TT_-J1-NANP_1_ vaccine poorly induced high-avidity r*Pf*CSP-specific IgG antibodies, even after the booster dose (Fig. 2e, D38). The avidity of the induced IgM antibodies was not tested separately, as we assume that their pentameric structure inherently provides high avidity for antigen binding^64^.

Interestingly, the induction of r*Pf*CSP-specific IgG and IgM antibodies exhibits a strong relationship with the number of NANP repeats targeted by the vaccine candidate, which conforms to a saturation curve model (Fig. 2f). Moreover, a strong linear correlation (Pearson *r* = 1) was observed between the induction rate of specific high-avidity IgG and the number of NANP repeats targeted by the vaccine candidates (Fig. 2f).

After challenge at day 38, parasitemia was monitored on a daily basis in all mice starting 4 days after parasite infection, using flow cytometry and checking for the parasite-derived GFP signal within the erythrocytes (Supplementary Fig. 2). We chose flow cytometry over the more commonly used Giemsa staining due to its comparable, and at low parasitemia levels, sometimes even superior sensitivity, as observed in previous studies^65^. Additionally, flow cytometry offers several practical advantages, including high-throughput capacity, faster analysis time, and the requirement for less extensive training or experience for the experimenter. Mice with measured parasitemia levels exceeding 0.1% were classified as infected. At the end of the study (18 days post infection), 88.89% (8 out of 9) of mice immunized with CuMV_TT_-NANP_19_ exhibited sterile protection, as they remained free of detectable asexual blood-stage parasites (Fig. 2g). In contrast, sterile protection was observed in 55.56% (5 out of 9) of mice immunized with CuMV_TT_-J2-NANP_6_, and in 11.11% (1 out of 9) of mice immunized with CuMV_TT_-J1-NANP_1_ (Fig. 2g). All mice receiving the CuMV_TT_ control vaccine or remaining naïve developed parasitemia 6–8 days following infection (Fig. 2g).

In summary, our findings did not reveal enhanced immunogenicity or improved protective efficacy of the vaccine candidates targeting the junction domain in combination with part of the cleavage site and the NANP repeat. However, we found a strong correlation between the number of NANP repeats displayed by the vaccine candidates and their capacity to elicit high-avidity r*Pf*CSP-specific IgG antibodies. Additionally, an increased number of targeted NANP repeats was associated with higher titers of *Pb/Pf*CSP- and r*Pf*CSP-specific IgG/IgM antibodies, a relationship that appears to follow a saturation curve model. Enhanced specific humoral responses resulted in improved protection against parasitemia following parasite infection.

### Optimized CuMV_TT_-based malaria vaccine candidates were successfully generated by expression in *E. coli*

**Fig. 3:**
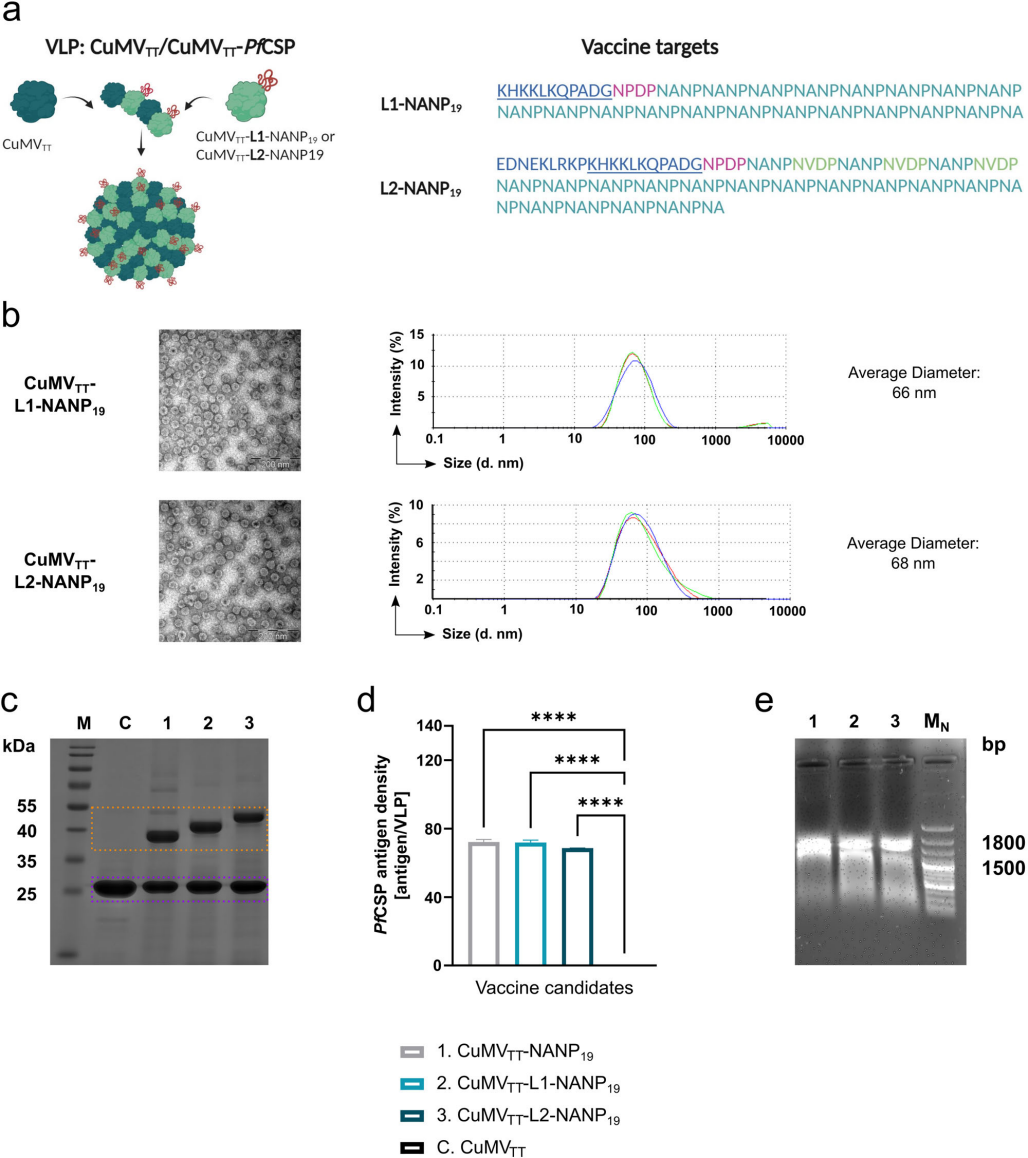
Design and characterization of optimized CuMV_TT_-based malaria vaccine candidates. **a** Schematic illustration of the design of optimized vaccine candidates. Vaccine candidates are based on CuMV_TT_-VLPs genetically fused to different amino acid sequences of *Pf*CSP (PlasmoDB: PF3D7_0304600). The sequences include KHKKLKQPADGNPDP followed by 19 NANP repeats (CuMV_TT_-L1-NANP_19_) and EDNEKLRKPKHKKLKQPADGNPDP followed by 19 NANP repeats interspersed with three single NVDP repeats (CuMV_TT_-L2-NANP_19_). Particles are expressed as mosaic VLPs, comprising CuMV_TT_ subunits fused to parts of *Pf*CSP and subunits that remain unfused. The scheme was created with BioRender.com. **b** TEM and DLS of vaccine candidates after expression in *E. coli*, scale bar 200 nm. **c** 12% SDS–PAGE of M. Protein Marker, C. CuMV_TT_, 1. CuMV_TT_-19 NANP, 2. CuMV_TT_-L1-NANP_19_, and 3. CuMV_TT_-L2-NANP_19_ after staining with coomassie. Protein bands resulting from the fusion of CuMV_TT_ to different amino acid sequences of *Pf*CSP are highlighted in orange, protein bands derived from CuMV_TT_ are marked in violet. **d** Antigen density of *Pf*CSP-derived amino acid sequences on the surface of the particles: 1. CuMV_TT_-NANP_19_, 2. CuMV_TT_-L1-NANP_19_, 3. CuMV_TT_-L2-NANP_19_, and C. CuMV_TT_. Antigen density was calculated by analyzing protein band intensities from SDS–PAGE depicted in (**c**) using FIJI image J software^22^. **e** Agarose gel analysis of 1. CuMV_TT_-NANP_19_, 2. CuMV_TT_-L1-NANP_19_, 3. CuMV_TT_-L2-NANP_19_, and M_N_. nucleic acid marker depicting the VLP encapsulated prokaryotic RNA. Statistical analyses were performed as follows: data are presented as mean ± SEM. In (**d**) one-way ANOVA with Tukey correction was used for comparison. Significance levels are denoted as follows: *p* < 0.0001 (****). *N* = 2.

Contrary to our initial expectations, our first experiments did not demonstrate an improved efficacy of vaccine candidates targeting the junction domain in combination with the NANP repeats. However, we identified a strong association between the number of NANP repeats displayed by the vaccine candidates and their immunogenicity as well as efficacy. Based on these findings, we designed two new vaccine candidates. The first candidate, CuMV_TT_-L1-NANP_19_, was designed to target the entire cleavage site, the NPDP junction domain, and 19 NANP repeats. The second candidate, CuMV_TT_-L2-NANP_19_, expanded on this by including the amino acids EDNEKLRKP located upstream of the cleavage side, as well as three additional NVDP repeats in between the NANP repeats (Fig. 3a). Both vaccine candidates were expressed in *E. coli* and purified as described above. TEM analysis confirmed the formation of VLPs for both candidates, showing the characteristic icosahedral geometry and *T* = 3 symmetry typical of CuMV_TT_-derived VLPs (Fig. 3b left)^46,58,61^. CuMV_TT_-L1-NANP_19_ had an average particle diameter of 66 nm, while CuMV_TT_-L2-NANP_19_ particles had an average diameter of 68 nm (Fig. 3b right). These particles were slightly larger than those of the CuMV_TT_-NANP_19_ vaccine (62 nm; Fig. 1b), a difference attributable to the size of the inserted sequence. Additionally, the single peak observed in DLS analysis indicated that these vaccine candidates were free of impurities and consisted of uniformly sized particles (Fig. 3b right). SDS–PAGE and FIJI image J software-based analysis revealed a similar incorporation rate of fused subunits into VLPs for both newly generated vaccine candidates as for CuMV_TT_-NANP_19_ (Fig. 3c, d). We also confirmed the presence of prokaryotic RNA within the particles of the optimized vaccine candidates by agarose gel electrophoresis (Fig. 3e).

### Immunogenicity and efficacy of the *Pf*CSP-targeting vaccine candidates are primarily driven by the *PfCSP* NANP repeat region

**Fig. 4:**
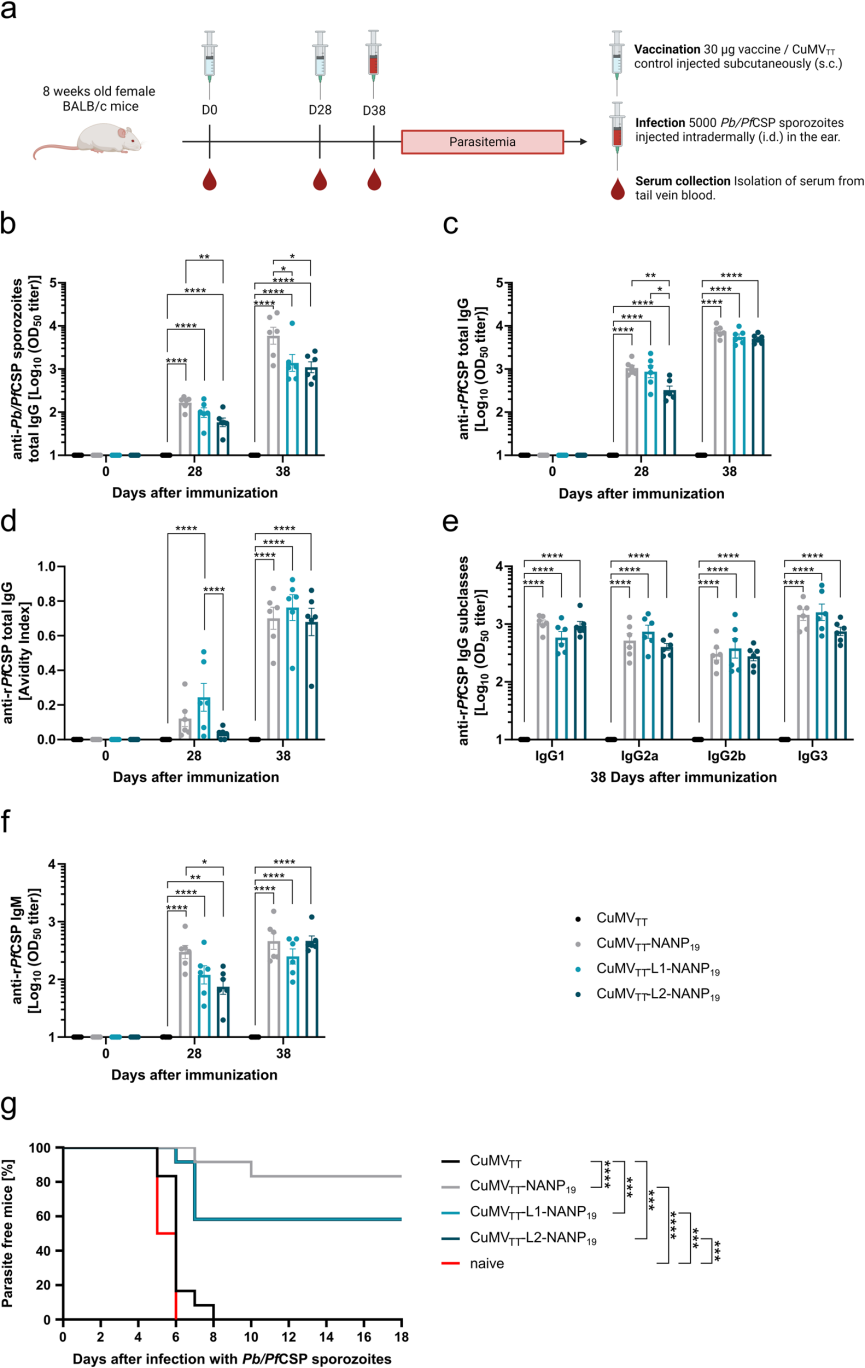
Immune responses and protective efficacy after vaccination with optimized CuMV_TT_-based malaria vaccine candidates. **a** Mouse model to evaluate the immunogenicity and protective efficacy of the optimized malaria vaccine candidates. 8-week-old female BALB/cOlaHsd mice were immunized on days 0 and 28 with 30 µg of the vaccine candidate, formulated in 100 µL PBS per mouse and administered s.c. On day 38, the mice were infected with 5000 *Pb/Pf*CSP sporozoites, delivered i.d. in the ear. Parasitemia was assessed daily by flow cytometry, checking for GFP-positive erythrocytes (*Pb/Pf*CSP infected). Blood samples were collected from the tail vein on days 0, 28, and 38, and serum was isolated for immunological analysis. The scheme was created with BioRender.com. **b** *Pb*/*Pf*CSP sporozoite-specific serum IgG measured by ELISA, Log_10_ OD_50_ is shown. **c** r*Pf*CSP-specific serum IgG measured by ELISA, Log_10_ OD_50_ is shown. **d** Avidity Index (proportion of high-avidity antibodies) of r*Pf*CSP-specific serum IgG measured by an adapted ELISA protocol using a 7 M urea washing step to remove low-avidity antibodies from the plate. **e** r*Pf*CSP-specific serum IgG1, IgG2a, IgG2b, and IgG3 measured by ELISA, Log_10_ OD_50_ is shown. **f** r*Pf*CSP-specific serum IgM measured by ELISA, Log_10_ OD_50_ is shown. **g** Parasite-free mice after i.d. injection of 5000 *Pb/Pf*CSP sporozoites. Statistical analyses were performed as follows: data are presented as mean ± SEM. In (**b**–**f**), one-way ANOVA with Tukey correction was used for comparisons. In (**g**), statistical significance was assessed using the Log-rank (Mantel–Cox) test. Significance levels are denoted as follows: *p* < 0.05 (*), *p* < 0.01 (**), *p* < 0.001 (***), *p* < 0.0001 (****). *N* = 6. One representative of two similar experiments is shown. Data of both experiments were combined in (**g**) (*N* = 12).

We tested the immunogenicity and the efficacy of the optimized vaccine candidates by using the same murine model of malaria and the same vaccination regiment as before. Briefly, 8-week-old female BALB/cOlaHsd mice were immunized s.c. either with 30 µg of the vaccine candidates or with 30 µg CuMV_TT_ control, both diluted in PBS, on days 0 and 28. 10 days after the booster injection, each mouse was infected with 5000 *Pb/Pf*CSP sporozoites via i.d. injection. Infection progression was monitored daily by flow cytometry, beginning 4 days post-sporozoite challenge, to detect the presence of asexual blood-stage parasites (Fig. 4a and Supplementary Fig. 3).

Serum samples were collected from immunized mice on days 0 (immunization), 28 (boost), and 38 (before sporozoite challenge) and analyzed by ELISA for whole *Pb/Pf*CSP sporozoites-specific IgG as well as for r*Pf*CSP-specific IgG and IgM. Notably, CuMV_TT_-NANP_19_ immunized mice displayed a significantly higher concentration of *Pb/Pf*CSP sporozoite-specific IgG antibodies compared to CuMV_TT_-L2-NANP_19_ immunized mice on both days 28 and 38. Following the booster injection (D38), CuMV_TT_-NANP_19_ immunized mice also showed higher levels of sporozoite-specific IgG compared to mice immunized with CuMV_TT_-L1-NANP_19_ (Fig. 4b). A similar pattern was observed for IgG antibody titers specific to the r*Pf*CSP protein on day 28. However, after the booster injection (D38), all vaccine candidates induced comparable levels of r*Pf*CSP-specific IgG (Fig. 4c). For all vaccinated groups we observed an increase in antibody titers following the booster injection, both for *Pb*/*Pf*CSP sporozoite- and r*Pf*CSP-specific IgG (Fig. 4b, c). Importantly, no *Pb/Pf*CSP sporozoite-specific or r*Pf*CSP-specific IgG antibodies were detected before immunization or in mice immunized with CuMV_TT_ control (Fig. 4b, c). On day 38, ~70% of the induced r*Pf*CSP-specific IgG antibodies displayed high avidity, regardless of the vaccine candidate used (Fig. 4d). Additionally, all three vaccine candidates induced a comparable and well-balanced IgG subclass response (Fig. 4e). r*Pf*CSP-specific IgM antibody titers evolved similarly to r*Pf*CSP-specific IgG titers and were equivalent between vaccine groups on day 38 (Fig. 4f).

**Fig. 5:**
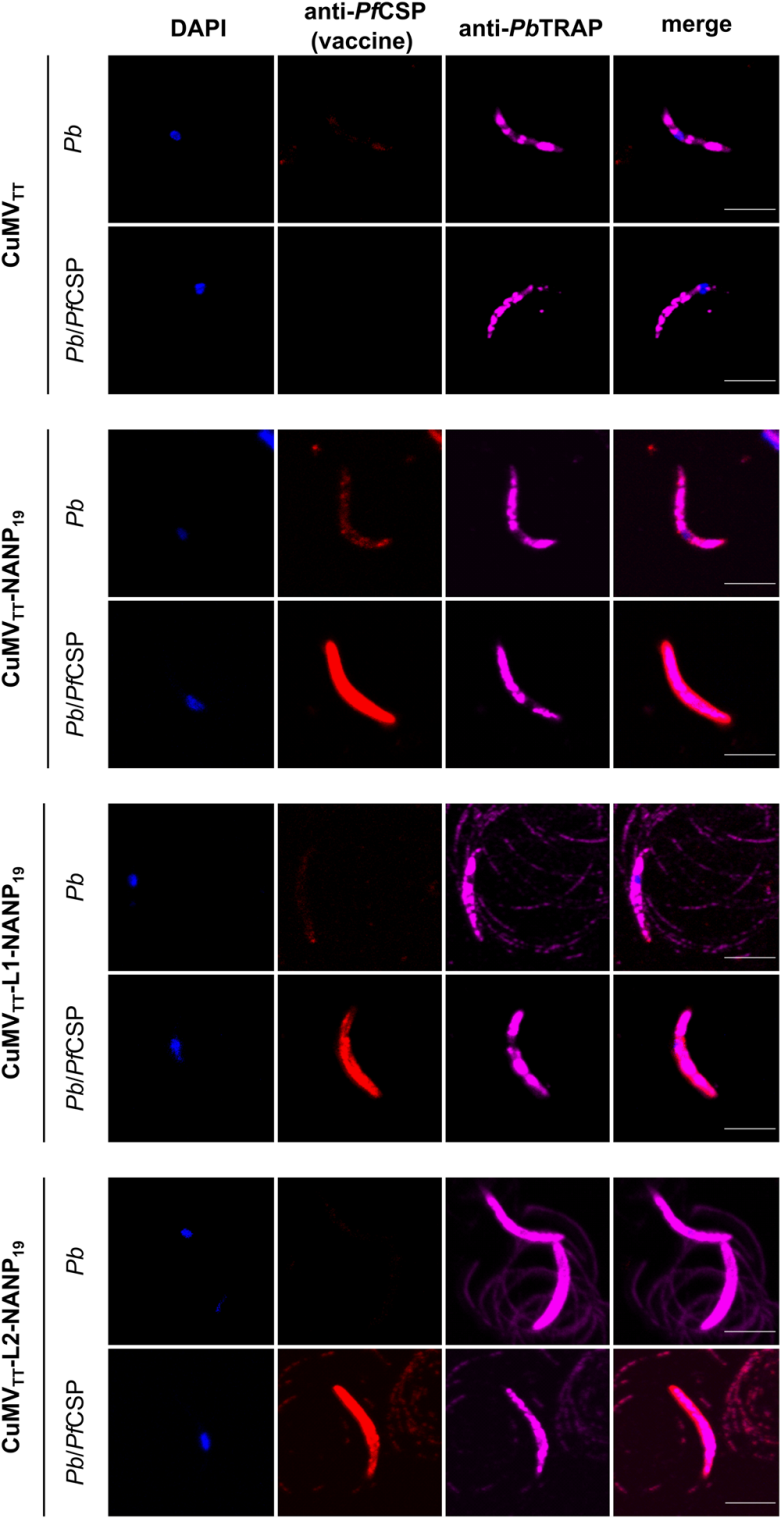
Binding capacity of vaccine candidate-induced IgG antibodies to *Pb*/*Pf*CSP sporozoites. Immunofluorescence assay showing the reactivity of serum from vaccinated mice (D38) with *Pb*/*Pf*CSP sporozoites. *Pb* sporozoites expressing endogenous *Pb*CSP (676 cl1 parental line) serve as a control. DAPI nucleic acid stain is depicted in blue, vaccine-induced IgG antibodies are depicted in red, and *Pb*TRAP-specific antibodies are depicted in purple. Scale bar 5 µm. One representative of two similar experiments is shown.

Furthermore, we conducted an in-depth examination of the antibodies’ binding capabilities to native *Pf*CSP present on the surface of sporozoites using immunofluorescence. Antibodies present in the sera collected on day 38 after immunization with CuMV_TT_-NANP_19_ or CuMV_TT_-L1/L2-NANP_19_ but not with CuMV_TT_ were capable of specifically binding to *Pf*CSP on the sporozoite surface. Minimal binding was observed to the endogenous *Pb*CSP expressed by control sporozoite (*Pb*) (Fig. 5). Despite the relatively low amino acid sequence identity between *Pb*CSP and *Pf*CSP, both proteins contain central repeat regions rich in proline and asparagine residues. Additionally, *Pb*CSP and *Pf*CSP share few conserved sequences at the N- and C-termini, including the KLKQP motif within the cleavage site^66^. These similarities may explain the minimal cross-reactivity observed. The binding capacity of the vaccine-induced antibodies was similar to that of a monoclonal antibody specifically binding to the repeat region of *Pf*CSP (Supplementary Fig. 3).

Naïve and CuMV_TT_ immunized mice exhibited detectable parasitemia 5–8 days post-challenge with *Pb/Pf*CSP sporozoites (Fig. 4g and Supplementary Fig. 3). In contrast, immunization with any of the tested malaria vaccine candidates significantly reduced the incidence of parasitemia. By the end of the experiment (18 days post-challenge), 83.33% (10 out of 12) of mice immunized with CuMV_TT_-NANP_19_ and 58.33% (7 out of 12) of mice immunized with either CuMV_TT_-L1-NANP_19_ or CuMV_TT_-L2-NANP_19_ remained free of asexual blood-stage parasites, indicating sterile protection (Fig. 4g and Supplementary Fig. 3). Overall, we observed comparable vaccine efficacy rates among all tested candidates.

In terms of eliciting specific IgM and IgG immune responses against r*Pf*CSP, both CuMV_TT_-L1-NANP_19_ and CuMV_TT_-L2-NANP_19_ demonstrated efficacy similar to CuMV_TT_-NANP_19_. All three candidates generated IgG antibodies that effectively bound to *Pf*CSP on the surface of *Pb/Pf*CSP sporozoites, leading to enhanced protection from parasitemia. Notably, CuMV_TT_-NANP_19_ induced the highest levels of *Pb/Pf*CSP sporozoite-specific IgG antibodies, which corresponded to a slightly lower incidence of parasitemia in this group following parasite challenge. Consequently, the incorporation of the junction domain, the NVDP minor repeats, and the cleavage site into the vaccine design did not yield enhanced immunogenicity or improved protection against the parasite. However, by including 19 NANP repeats in the design of the junction domain-targeting vaccine candidates, we successfully reinstated their immunogenic and protective characteristics. Therefore, our findings underscore the significance of the *Pf*CSP NANP repeat region, while suggesting that the junction domain, the NVDP minor repeats, and the cleavage site may not be as critical for *Pf*CSP-specific vaccine design.

### Evaluated vaccine candidates elicit limited NPDP-junction domain-specific antibody responses

The lack of enhanced vaccine efficacy upon incorporating the junction domain into the vaccine design could be due to several factors. It is possible that our vaccination strategy did not successfully induce NANP/NPDP-double-specific IgG antibodies or that these antibodies were outnumbered by NANP-mono-specific antibodies. Additionally, cross-reactivity could play a role. Given the high structural similarity, NANP repeat-specific antibodies might cross-react with the NVDP and NPDP motifs^42^.

**Fig. 6:**
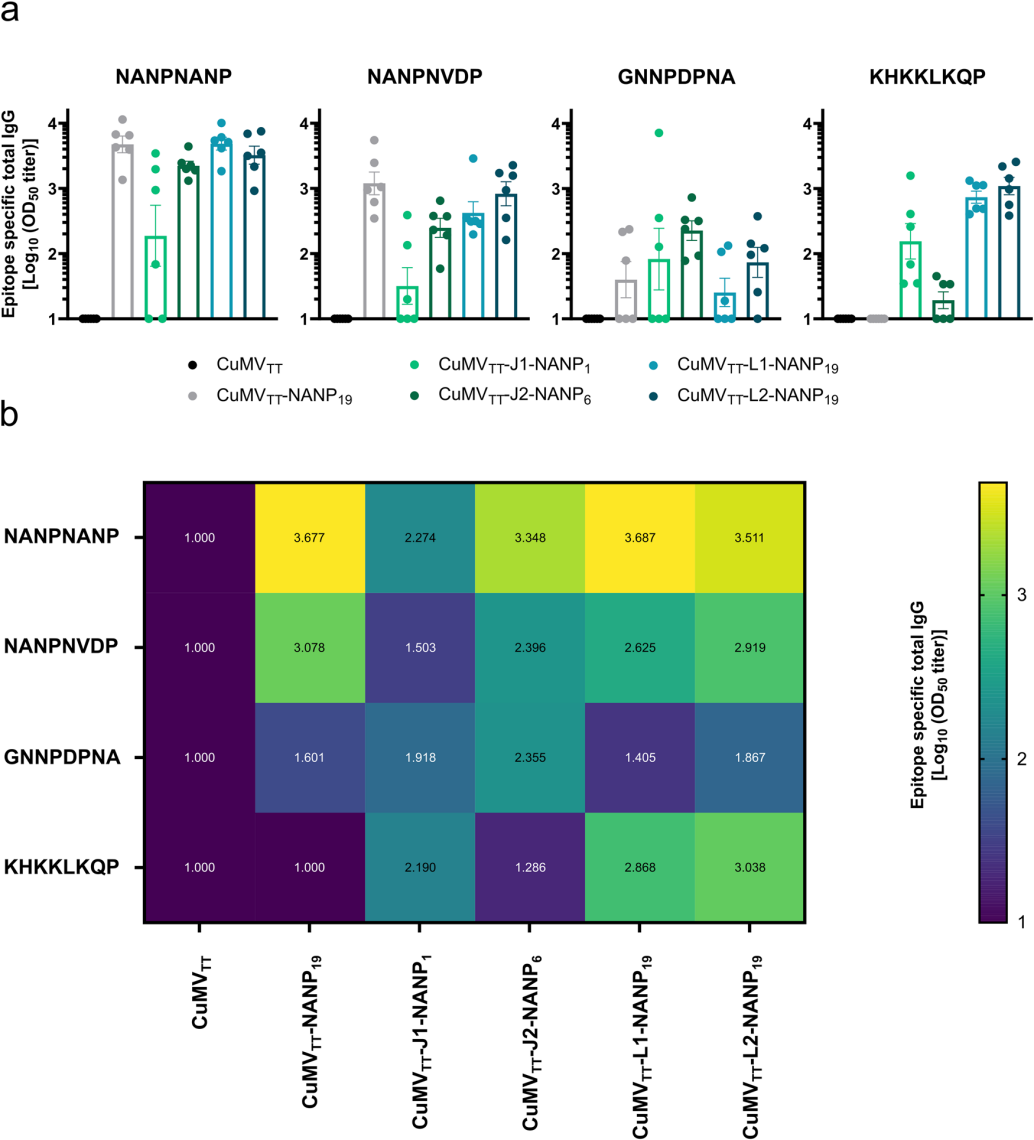
Epitope mapping of IgG antibodies induced by vaccine candidates. **a** Serum samples collected on day 38 analyzed by ELISA for epitope-specific IgG, Log_10_ OD_50_ is shown. Binding specificity to the following epitopes was assessed: 1. NANPNANP, 2. NANPNVDP, 3. GNNPDPNA, 4. KHKKLKQP. **b** Average epitope-specific serum IgG titer (Log_10_ OD_50_) displayed in a heat map. Data are presented as mean ± SEM. *N* = 6.

To examine this question in more detail, we performed epitope mapping of the IgG antibodies induced by the tested vaccine candidates. We assessed antibody specificity to the following epitopes: NANPNANP, NANPNVDP, GNNPDPNA, and KHKKLKQP using an ELISA-based approach. The induction of IgG antibodies binding to the NANPNANP epitope was associated with the number of repeats included in the vaccine design. CuMV_TT_-NANP_19_, CuMV_TT_-L1-NANP_19_, and CuMV_TT_-L2-NANP_19_ induced the highest levels of NANPNANP-specific antibodies, with all three vaccine candidates eliciting comparable responses. Slightly lower levels of NANPNANP-specific antibodies were detected following immunization with CuMV_TT_-J2-NANP_6_ and the lowest levels were observed for CuMV_TT_-J1-NANP_6_ (Fig. 6a, b). A similar pattern was observed for NANPNVDP-specific antibodies, although the overall antibody levels were lower (Fig. 6a, b). Interestingly, the most robust induction of GNNPDPNA-specific IgG was observed after immunization with CuMV_TT_-J2-NANP_6_, followed by CuMV_TT_-L2-NANP_19_. In mice immunized with CuMV_TT_-NANP_19_, CuMV_TT_-J1-NANP_1_, and CuMV_TT_-L1-NANP_19_, the GNNPDPNA-specific antibody responses were heterogeneous, with three non-responders and three responders in each group. Overall, immune responses targeting this junction domain epitope were relatively poor (Fig. 6a, b). Both vaccine candidates that included the entire KHKKLKQP epitope in their design, CuMV_TT_-L1-NANP_19_ and CuMV_TT_-L2-NANP_19_, induced the highest levels of antibodies binding to this epitope. Among the candidates targeting part of this epitope (KQP), CuMV_TT_-J1-NANP_1_ induced more antibodies compared to CuMV_TT_-J2-NANP_6_ (Fig. 6a, b). As expected, no KHKKLKQP-specific antibodies were observed in mice immunized with CuMV_TT_-NANP_19_. In general, no epitope-specific IgG was induced by CuMV_TT_ alone (Fig. 6a, b).

To conclude, the formation of NANPNANP- and NANPNVDP-binding antibodies is enhanced in vaccine candidates targeting the highest number of NANP repeats, here 19. Antibodies targeting the junction domain including epitope, GNNPDPNA, were in general weakly induced. Among all vaccine candidates tested, CuMV_TT_-J2-NANP_6_ produced the highest titers for this epitope. The CuMV_TT_-NANP_19_ vaccine candidate showed limited potential to induce cross-reactive antibodies binding to the NPDP junctional domain. Cleavage site-specific antibodies were preferentially observed after immunization with CuMV_TT_-L1-NANP_19_ and CuMV_TT_-L2-NANP_19_, the only vaccine candidates tested that included the entire cleavage site in their design.

## Discussion

Recent research has shown that *Pf*CSP-specific monoclonal antibodies neutralize *Pf* sporozoites most efficiently when recognizing both the NANP repeats and the N-terminal junction domain consisting of the four amino acids NPDP^30,41^. Notably, the currently registered malaria vaccines, RTS,S and R21, exclusively focus on the NANP repeat region, while disregarding the junction domain^13,19,20^.

To address this, our study aimed to assess if targeting both the *Pf*CSP NANP and NPDP regions enhances the effectiveness of malaria vaccine candidates. To investigate this, we have designed and produced five distinct vaccine candidates based on the established vaccine platform of CuMV_TT_-VLPs^54–59^. CuMV_TT_-NANP_19_ targets 19 of the *Pf*CSP NANP repeats and thus closely resembles the targeting sequence of the RTS,S and R21 vaccine, which includes 19 NANP repeats along with a few CD4^+^ T cell epitopes^19,20^. In consideration of the dominant role of the *Pf*CSP repeat-specific antibodies for the RTS,S as well as R21 efficacy, we excluded the T cell epitopes from the design of the vaccine candidates and focussed our experiments on antibody specificities^19,20,67^. CuMV_TT_-J1-NANP_1_ targets a minimal epitope consisting of part of the cleavage site, the junction domain, and one NANP repeat. CuMV_TT_-J2-NANP_6_ is a prolonged version of CuMV_TT_-J1-NANP_1_ additionally targeting five NANP repeats and two NVDP repeats. NVDP minor repeats also have been identified as a neutralizing epitope of *Pf*CSP^45^. The vaccine candidates CuMV_TT_-L1-NANP_19_ and CuMV_TT_-L2-NANP_19_ both target the complete cleavage site, the junction domain and 19 NANP repeats. The vaccine design of CuMV_TT_-L2-NANP_19_ further targets three additional NVDP repeats and a prolonged sequence upstream of the cleavage site.

The immunogenicity of CuMV_TT_-based vaccine candidates relies on three key factors: the integrity of the particles, the presence of encapsulated prokaryotic RNA, and the density of antigens displayed on the particle’s surface^21,22^. Only intact particles with the characteristic pathogen-associated structural patterns (PASPs), evidenced by correct particle symmetry and size can mimic a real virus and, in turn, stimulate the immune system^21,61,68^. The encapsulated RNA has an adjuvant-like role through its function as a TLR ligand^22^. Antigen density is of particular importance in that it influences BCR cross-linking and recruitment of complement, enhancing subsequent B cell activation and germinal center formation. Optimal BCR cross-linking occurs when antigens are spaced in a manner similar to that on the surface of real viruses with a spacing of 5–10 nm^47,69,70^. All the vaccine candidates examined in this study exhibited particles of icosahedral geometry with diameters falling within the range of 58–68 nm. Thus, all manufactured vaccine candidates possess the distinctive PASPs typically found in viral structures. Additionally, each vaccine VLP encapsulated prokaryotic RNA derived from *E. coli* and maintained a comparable antigen density on the surface. As such, we posit that any observed variations in immunogenicity and efficacy of the different vaccine candidates are a direct consequence of the differences in the target sequences and not the result of variations in particle integrity or epitope presentation.

As previously described for the RTS,S vaccine, we observed a clear correlation between the concentration as well as the avidity of IgG antibodies specific for *Pf*CSP and the efficacy of the vaccine candidates^12,26,42,67,71–73^. Interestingly, the induction of total IgG and especially high-avidity IgG specific for *Pf*CSP was dependent on the number of NANP repeats that were taken into account in the target sequence of the vaccine candidate. Also, the formation of *Pf*CSP-specific IgM was dependent on the number of NANP repeats displayed by the vaccine candidates. Earlier studies already pointed to the connection toward increasing immunogenicity and better protection by vaccines targeting more NANP repeats^74,75^. This is most likely due to a more efficient BCR cross-linking that occurs in the presence of high valency antigens, in this case in the presence of more NANP repeats^76^. However, this relation seems to be subject to a plateau effect as observed here by us and previously by others. For example, no difference in efficacy of the RTS,S vaccine was observed between subjects with a B cell epitope covering 37 or 44 NANP-NVDP repeats^77^. Furthermore, mice that were immunized with a truncated version of *Pf*CSP having 9 NANP repeats were even better protected from a mosquito bite challenge with *Pb/Pf*CSP sporozoites than mice that were immunized with *Pf*CSP covering 27 NANP repeats^78^. This may be counterintuitive, but can possibly be explained by the formation of cross-reactive antibodies. High-avidity binding of the long repeat regions to the BCR favors the formation of antibodies binding to the repeat region but hinders the formation of cross-reactive antibodies binding to the N- or C-terminal part of *Pf*CSP. However, these cross-reactive antibodies may also contribute to a status of protection and are hence an important part of a potent anti-malaria immune response^78^. Nevertheless, our vaccine candidates targeting the linker region and 1 or 6 NANP repeats failed to efficiently induce protective antibodies indicating the role of NANP repeats dominates over other regions. Our results are in line with the existing literature and point out the immunodominant role of the NANP repeats on the *Pf*CSP protein. Additionally, our findings highlight the importance of *Pf*CSP-specific high-avidity/affinity IgG antibodies for the induction of a protective humoral immune response against malaria.

Contrary to our initial hypothesis, we did not observe an enhanced vaccine efficacy when targeting both the junction domain and the cleavage site in addition to the NANP repeat region. In our first experiment, we even observed a decreased efficacy for the junction domain-specific vaccine candidates. However, this is most probably explained by the dependence of the efficacy on the number of NANP repeats included in the vaccine candidates, which we have already discussed. The inclusion of 19 NANP repeats into the target sequence effectively restored the immunogenicity of the junction domain-specific vaccine candidates, as evidenced by the consistent induction of *Pf*CSP-specific IgM, IgG, and high-avidity IgG antibodies. Nonetheless, the immune responses elicited were comparable to those induced by the CuMV_TT_-NANP19 vaccine. Notably, when it comes to *Pb/Pf*CSP sporozoites-specific IgG antibodies, even higher titers were observed in mice immunized with CuMV_TT_-NANP_19_. All tested vaccine candidates elicited IgG antibodies capable of binding *Pf*CSP, transgenically expressed on the surface of *Pb*/*Pf*CSP sporozoites, as confirmed by ELISA and immunofluorescence assay (IFA).

The robust immune response elicited by CuMV_TT_-NANP_19_, as well as the junction domain-targeting vaccine candidates CuMV_TT_-L1-NANP_19_ and CuMV_TT_-L2-NANP_19_, has translated into significantly extended survival rates following parasite infection. The highest level of protection was observed in mice vaccinated with CuMV_TT_-NANP_19_, which may be a direct consequence of the elevated *Pb/Pf*CSP-specific IgG titers detected in these mice. *Pf*CSP-specific antibodies confer protection against malaria not only by neutralizing sporozoites but also by facilitating Fc-mediated effector functions such as opsonization for phagocytosis, activation of the complement system, or NK cell-mediated cytotoxicity^37–40^.

The absence of enhanced vaccine efficacy upon incorporating the junction domain into the vaccine design prompted us to investigate the binding specificity of the induced antibodies in more detail. To this end, we carried out a binding analysis to the following epitopes: NANPNANP, NANPNVDP, GNNPDPNA, and KHKKLKQP.

Interestingly, among the vaccine candidates incorporating the NPDP junction domain in their target sequence, CuMV_TT_-J2-NANP_6_—which targets the junction domain in combination with six NANP and two NVDP repeats—elicited the highest titers specific to the junction domain including epitope GNNPDPNA. A previous study has shown that the high-avidity binding of B cells to the immunodominant NANP repeat regions of *Pf*CSP, which is enhanced by multiple NANP repeats in the target site, can suppress immune responses to subdominant epitopes present at the N or C terminus, such as NPDP for example^78^. This might explain why both vaccine candidates targeting the junction domain along with 19 NANP repeats induced fewer NPDP-specific antibodies than the CuMV_TT_-J2-NANP_6_ vaccine candidate. Following this logic, one would expect CuMV_TT_-J1-NANP_1_, which targets only 1 NANP repeat adjacent to NPDP, to induce the most NPDP-specific antibodies. However, this was not observed here.

Given the high structural similarity, NANP repeat-specific antibodies can cross-react with the NVDP and NPDP motifs^42^. Consequently, targeting the *Pf*CSP NANP repeat region through vaccination may induce antibodies capable of binding to NANP, NPDP, and NVDP motifs. However, in our study, we observed limited numbers of antibodies binding to the junction domain-targeting epitope after immunization with the CuMV_TT_-NANP_19_ vaccine candidate, which exclusively targets the central repeat region. Furthermore, the induction of NANPNVDP-specific antibodies was weaker compared to NANPNANP-specific antibodies across all vaccine candidate groups, indicating a relatively low potential for cross-reactivity. It has been shown that affinity maturation of NANP-specific antibodies correlates with the cross-reactive binding properties of these antibodies^42^. Although we observed a robust induction of *Pf*CSP-specific high-avidity/affinity IgG antibodies following immunization with CuMV_TT_-NANP_19_, the cross-reactive potential of these antibodies was limited.

The vaccine candidates that demonstrated the highest protection rates against parasitemia were those incorporating 19 NANP repeats in their target sequence (CuMV_TT_-NANP_19_, CuMV_TT_-L1-NANP_19_, CuMV_TT_-L2-NANP_19_). These candidates also elicited the highest levels of NANPNANP-specific antibodies.

Vaccination induces a polyclonal antibody response, whose overall efficacy depends on the collective efficacy of all induced antibodies and thus differs fundamentally from monoclonal antibodies^79^. This aspect must be considered when comparing our results with studies involving monoclonal antibodies. The vaccines tested here predominantly induced antibodies targeting the NANP repeats, which we found to be the primary mediators of protection against parasitemia. In contrast, antibodies predominantly targeting the junction domain or NVDP minor repeats were rather weakly induced. This does not imply that antibodies specific to these regions are less potent. However, it seems that inducing strongly protective antibody responses against these regions through vaccination is more challenging. Therefore, alternative vaccine designs, beyond those tested here, may be necessary to generate high antibody titers specifically targeting these regions.

Although antibodies against the junction domain or the NVDP minor repeats may hold potential, our data do not obviously support the inclusion of these regions into the design of novel *Pf*CSP-targeting vaccines. It appears more critical that vaccination induces a robust response of high-affinity NANP-specific antibodies. This observation aligns with the findings from a previous study, that observed NANP repeats presented on the surface of woodchuck hepatitis virus core antigen (WHcAg)-VLPs are sufficient to induce antibodies that confer sterile protection from a challenge with *Pb* sporozoites expressing *Pf*CSP when passively transferred^80^.

Two recent studies have extensively investigated the potential of targeting the binding sites of the potent monoclonal antibodies CIS43 (NPDPNANPNVDPNAN) and L9 (NANPNVDPNANPNVD) for the development of improved malaria vaccines. In these studies, the target sequences for both CIS43 and L9 were chemically conjugated to Qβ-VLPs. Both vaccine candidates demonstrated high immunogenicity in mice, particularly when co-formulated with the Advax-3 adjuvant, a combination of the TLR9 ligand CpG oligonucleotide and aluminum hydroxide. Targeting the CIS43 binding site not only induced antibodies specific to this site but also to the repeat region in general, albeit to a somewhat lesser extent. While both candidates were able to partially inhibit hepatocyte infection upon challenge with transgenic *Pb* sporozoites expressing the *Pf*CSP, sterile protection was only observed for the L9-targeting vaccine candidate. Nonetheless, both vaccines induced *Pf*CSP-specific antibody titers that remained stable for over 6 months (L9) or even more than 2 years (CIS43)—a significant achievement considering the short-lived nature of the RTS,S vaccine^26,81,82^.

Addressing a similar question as our study, these publications did not include a vaccine candidate that exclusively targets the NANP repeats, as we did here. It would be intriguing to investigate whether Qβ-VLPs exclusively displaying NANP repeats would result in comparable immunogenicity and protection, as our data suggests. Moreover, it remains to be seen whether NANP-specific vaccine candidates could elicit a similarly long-lived immune response as those targeting the junction domain. This particular aspect was neither addressed in our study nor in the aforementioned studies. A definitive conclusion on this matter would, of course, require that the vaccine candidates differ solely in their target epitopes and not in other factors such as the VLP or formulation.

*Pf*CSP-specific antibodies have been identified as pivotal contributors to malaria protection following RTS,S vaccination^12,26,42,67,71–73^. However, also *Pf*CSP-specific CD4^+^ T cells may play a significant role for the vaccine efficacy of RTS,S as they assist the antibody formation and stimulate cytokine production^20,67^. We did not rely on such T cell epitopes, as CuMV_TT_ with the TT epitope provide ample T cell help for induction of *Pf*CSP-specific IgG. Although RTS,S, and the here-tested CuMV_TT_-based malaria vaccine candidates rely both on mosaic VLPs, they significantly differ in terms of different aspects. RTS,S/R21 are based on hepatitis B-derived particles that are expressed in yeast, whereas our vaccine candidates rely on CuMVVLPs that are obtained upon expression in *E. coli*. Furthermore, the CuMV_TT_ platform includes a universal T cell epitope derived from the tetanus toxin and particles encapsulate prokaryotic RNA, which acts as TLR ligands. The contribution of encapsulated RNA in enhancing immunogenicity and protective efficacy has been previously demonstrated by us for the CuMV_TT_-based VLP Peanut vaccine^22^. Consequently, CuMV_TT_-encapsulated RNA serves as an additional immunological stimulus, akin to adjuvants, and most probably also contributes to the efficacy of the vaccine candidates evaluated here. All vaccine candidates tested in this study were formulated in PBS, without adjuvants. While we cannot rule out the possibility that adjuvants might have further enhanced immunogenicity, the high effectiveness of the vaccine candidates—leading to sterile protection in some cases—did not necessitate their use. However, a VLP-based vaccine targeting the thrombospondin-related adhesive protein (TRAP) of *Plasmodium vivax* demonstrated increased efficacy when formulated with the adjuvant Microcrystalline Tyrosine (MCT®)^83^.

In summary, CuMV_TT_-VLPs turned out to be a potent platform for *Pf*CSP-targeting malaria vaccine candidates. The efficacy of these vaccine candidates was dependent on *Pf*CSP-specific IgG and IgM antibodies. In particular, *Pf*CSP-specific high-avidity/affinity IgG antibodies were crucial for malaria protection. The induction rate of such high-avidity/affinity IgG antibodies but also the overall concentration of *Pf*CSP-specific antibodies positively correlated with the number of NANP repeats considered in the design of the vaccine candidates. In contrast to our hypothesis, we observed no improvement in vaccine efficacy by targeting the *Pf*CSP NPDP junction domain, the NVDP repeats or the cleavage site in addition to the NANP repeats.

## Methods

### Mice

In vivo experiments were performed using 8-week-old female BALB/cOlaHsd mice purchased from Envigo (Amsterdam, the Netherlands). Animal procedures were performed in accordance with the Swiss Animal Act (455.109.1—September 2008, 5th) at the DBMR of the University of Bern. All animal experiments were conducted by protocols approved by the Cantonal Veterinary Office Bern, Switzerland (License: BE80/2021).

To generate sporozoites, animal experiments were conducted in strict accordance with the guidelines of the Swiss Tierschutzgesetz (TSchG; Animal Rights Laws) at the Institute of Cell Biology and approved by the ethical committee of the University of Bern (Permit Number: BE98/19 and BE118/2022). Mice were kept in specific pathogen-free conditions and subjected to regular pathogen monitoring by sentinel screening. Moreover, mice were kept in individually ventilated cages furnished with autoclaved aspen woodchip, a mouse house, and paper tissue at 21 ± 2 °C under a 12:12 h light–dark cycle at a relative humidity of 55 ± 10%. Animals were fed with a commercially prepared autoclaved dry rodent diet and water, both available ad libitum. The health of animals was monitored daily by visual health checks and the parasitemia of infected animals was determined by flow cytometry.

### Production and purification of vaccine candidates

Manufacturing of CuMV_TT_ control was described in detail in Zeltins et al.^46^. VLP-based malaria vaccine candidates were produced as mosaic particles consistent of unmodified CuMV_TT_ and modified CuMV_TT_ subunits genetically fused to sequences of the *Pf*CSP, similar as described for VLP Peanut^58^.

Briefly, the CMV-Ntt830 gene was inserted under the T7 promotor into the pET Duet-1 vector (Novagen, Germany, Cat. 71146). The product was amplified by PCR and cloned into the pTZ57R/T vector using the InsTAclone PCR Cloning Kit (Fermentas, Lithuania; Cat. K1214) in *E. coli* XL1-Blue cells (Agilent, USA, Cat. 200249). Correct plasmid clone formation was verified using the BigDye Cycle Sequencing Kit (Thermo Fisher Scientific, USA; Cat. 4337449) as well as the ABI Prism 3100 Genetic Analyzer (Applied Biosystems, USA). Plasmids were treated with HindII restriction enzyme (Thermo Fisher Scientific, USA; Cat. ER0502), Klenow enzyme (Thermo Fisher Scientific, USA; Cat. EP0422) Klenow enzyme as well as the Ndel restrictase (Thermo Fisher Scientific, USA; Cat. ER0581) and obtained fragment sub-cloned into the Ndel/EcoRV sites of the pET Duet-1 vector (Novagen, Germany, Cat. 71146) resulting in pETDu-CMV-Ntt830. The CMVB3d-*Pf*CSP gene was excision from pACYCDu-CMVB3d-*Pf*CSP (pACYCDu: Novagen, Germany, Cat. 71147) using NcoI (Thermo Fisher Scientific, USA; Cat. ER0572) and HindIII (Thermo Fisher Scientific, USA; Cat. ER0502) enzymes and inserted under the T7 promotor of pETDu-CMV-Ntt830. Restriction enzyme analysis was performed to identify correct clones (pETDu-CMVB3d-*Pf*CSP-CMVtt830).

*E. coli* C2566 (New England Biolabs, USA) cells were transformed with the plasmid pETDu-CMVB3d-*Pf*CSP-CMVtt830. Successfully transformed *E. coli* cells were grown in 2TY medium (Trypton 1.6%, yeast extract 1%, 0.5% NaCl, 0.1% glucose) containing ampicillin (50 mg/L) on a rotary shaker (10 rpm, 30 °C) to OD_600_ absorption of 0.8. Cultures were induced with 0.2 mM IPTG and the medium was supplemented with 5 mM MgCl_2_. Incubation was continued on the rotary shaker (10 rpm, 20 °C, 18 h), and resulting biomass was collected by centrifugation (15,000 × *g*, 4 °C, 10 min).

Cells were disrupted by sonication for 16 min on ice (Hielscher 200, power 70%, pulse 50%) in the following buffer: 20 mM Tris, 5 mM EDTA, 5 mM Et-SH, 5% glycerol, 10% sucrose (pH 8.0); 0.5% TX-100 was added and solution was rotated (10 rpm) overnight at 4 °C.

The solution was centrifuged for 10 min at 10,000 rpm (Eppendorf 5804) and soluble fraction was loaded on a 20–60% sucrose gradient in the following buffer: 20 mM Tris, 2 mM EDTA, 5% glycerol, 0.5% TX-100. Centrifugation was performed for 6 h at 18 °C at a speed of 25,500 rpm (Beckman Coulter SW32). Fractions were analyzed by SDS–PAGE and VLP containing fractions sedimented by centrifugation (50,000 rpm, 4 h, 4 °C; Beckman Coulter 70Ti). Pellet was dissolved in 20 mM Tris, 2 mM EDTA (pH 8.0) and loaded on a 30% sucrose cushion containing 20 mM Tris, 2 mM EDTA, 5% glycerol, 0.5% TX-100 (72,000 rpm, 1 h, 4 °C; Beckman Coulter TLA-100.3). Obtained VLPs were solubilized in (20 mM TRIS, 2 mM EDTA, pH 8.0) and analyzed by SDS–PAGE, agarose gel, TEM, and DLS (Figs. 1 and 3). Endotoxin levels for all vaccine candidates were below 100 EU per injection.

### SDS–PAGE analysis

15 µL sample (1 mg/mL) was mixed with 3 µL reducing buffer (Thermo Scientific, Cat. 39000), heated for 5 min at 95 °C and then loaded onto a 12% SDS–PAGE with a 4% stacking gel. 6 µL protein ladder (Thermo Scientific, Cat. 26616) were loaded and the gel was run at 70 V in the following buffer: Tris (hydroxymethyl)-aminoethane 2.5 mM, glycine 25 mM, SDS 0.01%. Protein bands were stained with InstantBlue® Coomassie Protein Stain (Abcam, Cat. Ab119211) and a gel picture was taken with Azure Biosystems c300 using the visible channel, auto-exposure time. Incorporation rate of *Pf*CSP-CuMV_TT_ fused subunits into VLPs was determined as previously described^22^. Briefly, protein band intensities were quantified with FIJI Image J software v1.53c^84^. The *Pf*CSP-CuMV_TT_-derived protein band intensity was divided by the corresponding CuMV_TT_-derived protein band intensity for each sample. The obtained ratio was corrected for the differed molecular masses of the proteins and multiplied by 180, the total number of subunits of CuMV_TT_^46^.

### Agarose gel

15 µL sample (1 mg/mL) was mixed with 2.5 µL loading dye (New England BioLabs, Cat. B7024S) and loaded with ladder (Thermo Scientific, Cat. SM0242) onto a 2% agarose (BioConcept, 7-01P02-R) gel, run in Tris–borate–EDTA buffer at 50 V. Gel picture was taken with Azure Biosystems c300 using the UV302 channel, auto-exposure time.

### Transmission electron microscopy (TEM)

VLP formation was confirmed for each vaccine sample produced by TEM (Philips CM12 EM). For this purpose, sample grids were glow discharged and 10 μL of VLP sample (1 mg/mL) was added for 30 s. Afterward, Grids were washed three times with ddH_2_O and negatively stained with 5 μL of 5% uranyl acetate for 30 s. Excess of uranyl acetate was removed by pipetting. Grids were air-dried for 10 min and images were taken with 84,000× and 110,000× magnification.

### Dynamic light scattering (DLS)

Average VLP particle size in vaccine batches was measured by DLS using the Zetasizer Nano ZS instrument (Malvern Panalytical Ltd., UK), and measurements were evaluated with DTS software v. 6.32 (Malvern Panalytical Ltd., UK). 3 consecutive measurements were performed per sample.

### Vaccination regimen for naïve mice

Mice were immunized subcutaneously (s.c.) with 30 µg vaccine candidate or CuMV_TT_ control in 100 µL PBS using an insulin syringe (B Braun Omnican^®^, Cat. 9151133S). 28 days post prime a booster injection with an equal dose was given. Serum samples were collected the day of prime (D0), the day of boost (D28, before vaccination), and 10 days post boost (D38, before sporozoite challenge) via tail-vein bleeding, and serum was isolated using BD Microtainer^®^ Collection Tube (BD Biosciences, Cat. 365967).

### Parasite lines

For the challenge and IFA experiments a chimeric *Pb* parasite line in which the endogenous CSP gene was replaced with the *Pf* counterpart has been used. This chimeric parasite line was generated by Triller et al. and obtained from the Rodent Malaria genetically modified Parasites database (RMgmDB; ; 2257 cl2, RMgm 4110)^31^. Brief description of the parasite line: the endogenous *Pb* CSP was replaced with the *Pf* CSP in the parental line (676 cl1, RMgm-29; Janse et al.^85^). This parental line expresses a fusion protein of GFP (gfp-mu3) and Luciferase Firefly (LucIAV) under the expression of the *eef1a* promoter at the *230p* silent locus^85^. The resulting line, *Pb/Pf*CSP-GFP-Luc, has been referred to as *Pb/Pf*CSP in our study. Importantly, these chimeric *Pb/Pf*CSP parasites exhibited robust sporozoite development in mosquitoes, with successful expression of *Pf*CSP on the sporozoite surface. Furthermore, the in vivo infectivity of *Pb/Pf*CSP parasites in wild-type mice resembled that of parasites from the parental *Pb* line^31,86,87^. For IFA, the parental line (676 cl1, RMgm-29; Janse et al.^85^) has been used as a control.

### Mosquito, parasite maintenance, and salivary gland sporozoites dissection

#### Mosquito maintenance

*Anopheles stephensi* (*A. stephensi*) mosquitoes were bred at 28 °C, 80% humidity, and a 12 h light–dark cycle in an incubator (MLR-352H SANYO, Japan) in the insectarium at the Institute of Cell Biology of the University of Bern. Mosquitoes were daily fed with cotton pads containing 8% fructose solution supplemented with 0.2% PABA (Sigma-Aldrich, Cat. No. 100536) and female mosquitoes were fed with human blood once a week to allow them to lay eggs. The next day, eggs were collected, washed once with 70% ethanol, and twice with water before being added to a metal bowl with a drop of about 10 µl of NobilFluid Artemia (JBL 33801 Germany), where they developed into larvae. After 2 days in the metal bowl, the larvae were transferred into a water-containing plastic bowl and fed with grounded Tetra TabiMin complete food tablets (Olibetta, 400080, Germany). After 7 days, pupae were collected and put in a stock cage, where they were kept at 27 °C and 80% humidity until hatching. Adult mosquitoes were fed with 8% fructose supplemented with 0.2% PABA (Sigma-Aldrich, Cat. No. 100536). After 2 days, female *A. stephensi* mosquitoes were ready to be infected with *Pb/PfCSP* parasites and could therefore be collected from the stock cage. After feeding, mosquitoes were kept at 20 °C.

#### Parasite maintenance

Female BALB/cOlaHsd mice (6–8 weeks; Janvier Laboratories, France) were used to maintain parasites and for mosquito feeding with parasites. Mice were injected with *Pb*-infected blood via an intraperitoneal route and when parasitemia reached 2%–5%, mice were euthanized in a CO_2_ chamber and parasites isolated following exsanguination and the infected blood was intravenously passaged into a naïve mouse for further mosquito feeding. Upon reaching a parasitemia of 5%–7% and verifying for gametocytes presence (0.5%–1%), mice were anesthetized with a terminal dose of ketamine: xylazine, and when no longer reacting to touch stimulus was placed on a cage of ~100–150 mosquitoes for 45 min at 20 °C. Infected mosquitoes were kept at 20 °C and 80% humidity and fed with 8% fructose, containing 0.2% PABA for 16 and up to 26 days for salivary gland sporozoite dissection.

#### Salivary gland sporozoites dissection

Mosquitoes were aspired with a vacuum (Fulton U.S. MX-991/U, Hausherrs Machine Works, USA), anesthetized with chloroform and stored during dissection on ice. Before dissection, mosquitoes were dipped into PBS, 70% ethanol, and again into PBS to be placed on a slide, and salivary glands were extracted under the binocular (SZX 10 Olympus, Japan) with forceps and collected in a tube containing 50 μL of non-complement Iscove’s Modified Dulbecco’s Medium (IMDM). Salivary glands were homogenized (Pellet pestles Cordless Motor, Sigma-Aldrich, USA) and counted with a Neubauer counting chamber (VWR International GmbH, Switzerland) before being mixed with PBS and i.d. injected into mice.

### Challenge of vaccinated mice with *Pb*/*Pf*CSP sporozoites

Previously immunized mice were challenged with *Pb*/*Pf*CSP sporozoites 10 days after the booster injection. To this end, mice were inoculated with 5000 sporozoites given in 20 µL PBS i.d. (10 µL into each ear) by using an insulin syringe (B Braun Omnican^®^, Cat. 9151133S). Parasitemia was monitored daily by flow cytometry. For this purpose, 1 µL blood from the tail vein was mixed with 500 µL PBS supplemented with 2% FBS and 100 mM EDTA. *Pb/PfCSP*-infected erythrocytes of blood samples were analyzed by flow cytometry (CytoFLEX, Beckman Coulter) for GFP positivity (*Pb*/*Pf*CSP parasites express GFP as described above). Data were collected using CytExpert™ Software, version 2.6. Data were analyzed by using FlowJo™ Software, version v10.7 (BD Biosciences). Mice having a measured parasitemia >0.1% were considered infected. Mice having a measured parasitemia >2% were euthanized (termination criterion).

### Production and purification of recombinant *Pf*CSP (r*Pf*CSP)

The production of recombinant *Pf*CSP followed the procedure previously described^41^. Briefly, *Pf*CSP sequence of the Pf3D7 strain (PlasmoDB ID: PF3D7_0304600.1) was codon-optimized for the gene expression in mammalian cells (GenScript, USA). The first 20 leader amino acids were changed to a mammalian secretory signal peptide derived from the modified bovine lactine (MDSKGSSQ KGSRLLLLLVVSNLLLPQGVLA) and the GPI-anchor residues 376–397 were replaced with a GSG-linker followed by an 8x-histidine-tag. The gene construct was cloned into a pcDNA3.1(+) expression vector under the control of the T7 promotor (GenScript, USA). r*Pf*CSP was expressed by using the Expi293™ expression kit (Thermo Scientific, Cat. A14635) according to the manufacturer’s protocol. The supernatant was harvested 5 days post-transfection and dialyzed to PBS using Spectra/Por™ dialysis membrane (Fisher Scientific, Cat. 08-667B). Protein purification was carried out by affinity chromatography using His-Trap HP columns (Cytiva, Cat. 17524701) and the Äkta go™ (Cytiva, USA) purification system. Protein concentration was determined using Pierce™ BCA Protein Assay Kit (Thermo Scientific, Cat. 23225). Correct expression was verified by SDS–PAGE (previously described here) and western blot analysis. For western blot, r*Pf*CSP was separated on SDS–PAGE, and protein bands were transferred onto a 0.2 µM nitrocellulose membrane (BioRad, Cat. 1704158) using the Trans-Blot® Turbo™ Transfer System (BioRad, USA) and further processed by using the iBind™ Flex Western Device (Invitrogen, USA) according to the manufacturer’s instructions. His-tag-carrying protein bands were detected with the HRP anti-His Tag antibody (BioLegend, Cat. 652504), 1:1000 diluted. *Pf*CSP protein bands were detected with the anti-*Pf*CSP antibody (obtained through BEI Resources, NIAID, NIH: Monoclonal Anti-*Pf* CSP, Clone 2A10 (produced in vitro), MRA-183A, contributed by Elizabeth Nardin) used as primary antibody at 1:2000 dilution and HRP anti-mouse IgG (Jackson ImmunoResearch, Cat. 115-035-071) at 1:1000 dilution as secondary antibody. Membrane was developed by using SuperSignal™ West Pico PLUS Chemiluminescent Substrate (Thermo Scientific, Cat. 34579) and the image was obtained with Azure Biosystem c300, 5 s exposure time.

### Enzyme-linked immunosorbent assay (ELISA)

*Pb/Pf*CSP sporozoite and r*Pf*CSP-specific IgG antibodies in serum of immunized mice were detected by ELISA. For this, ELISA plates (CORNING, Cat. 3690) were coated with 50 µL *Pb/Pf*CSP sporozoites diluted in PBS (30 sporozoites/µL) or 50 µL r*Pf*CSP diluted in PBS (1 µg/mL) overnight at 4 °C. ELISA plates were washed four times with PBS using the Microplate washer (BioTek 405 TS Microplate Washer, Agilent), 100 μL/well PBS. Unspecific binding was prevented by treating plates with PBS-Casein 0.15% for 2 h at room temperature (100 μL/well). Plates were flicked and mouse serum 1:20 pre-diluted in PBS-Casein 0.15% was added to the top row of the plate. A 1:3 serial dilution in PBS-Casein 0.15% was performed and plates were incubated for 1.5 h at room temperature (total volume of 50 µL/well). Subsequently, plates were washed with PBS-0.01% Tween_20_ for four times with Microplate washer (100 μL/well), and HRP goat anti-mouse IgG (Jackson ImmunoResearch, Cat. 115-035-071) was added at 1:1000 dilution in PBS-Casein 0.15% (50 µL/well), plates were incubated for 1 h at room temperature. Plates were washed with PBS-0.01% Tween_20_ for four times with Microplate washer (100 μL/well) and developing solution (30 mM citrate buffer including 5% tetramethylbenzidine and 1.5‰ H_2_O_2_) was added (50 μL/well). The color reaction was stopped by the addition of 1 M H_2_SO_4_ (50 μL/well) and absorbance at 450 nm (OD_450_) was read with BioTek Cytation 5 imaging reader. Half-maximal antibody titers (OD_50_) were defined as the reciprocal of the dilution leading to half of the OD measured at saturation.

r*Pf*CSP-specific IgG subclass and IgM antibodies were determined following the same protocol but using a different detection antibody: HRP rat anti-mouse IgG1 (BD Biosciences, Cat. 559626, 1:1000 dilution), HRP rat anti-mouse IgG2a (BD Biosciences, Cat. 553391, 1:1000 dilution), HRP goat anti-mouse IgG2b (Invitrogen, Cat. M32407, 1:1000 dilution), HRP goat anti-mouse IgG3 (Southern BioTech, Cat. 1101-05, 1:1000 dilution), HRP goat anti-mouse IgM (Jackson ImmunoResearch, Cat. 115-035-075, 1:1000 dilution).

High-avidity IgG antibodies specific for r*Pf*CSP were determined by a previously described protocol^63^. For this, the normal ELISA protocol was extended by an additional washing step. Two similarly coated plates incubated with the same sera were washed upon sera incubation either three times for 5 min with PBS-0.01%Tween_20_ or three times for 5 min with 7 M urea in PBS-0.01% Tween_20_ (50 µL/well). Afterwards, detection antibody was added and the above-described protocol continued. The AI was calculated by AIx = (OD_450_ (dilution ×) urea treated plate)/(OD_450_ (dilution ×) non-urea treated plate).

### Epitope mapping

To map the epitopes of IgG antibodies induced by the different malaria vaccine candidates, an ELISA-based method was employed. ELISA plates (CORNING, Cat. 3690) were coated with 50 µL of BSA-conjugated peptides diluted in PBS (5 µg/mL) and incubated overnight at 4 °C. The peptides used were as follows: H-CKHKKLKQP-NH2, H-CDGNPDPNA-NH2, H-CNANPNVDP-NH2, and H-CNANPNANP-NH2, all conjugated to BSA via a cysteine residue and purchased from BIOSYNTH®. Following peptide coating, the standard ELISA protocol was carried out as previously described.

### Immunofluorescence assay (IFA)

Salivary gland sporozoites were dissected as previously described. Collected sporozoites were resuspended in minimum essential medium (MEM; BioConcept, 1-31F01-l) supplemented with 1% l-glutamine (Bioconcept, 5-10K00-H) and 1% penicillin–streptomycin (Bioconcept, 4-01F00-H) and complemented with 10% heat-inactivated fetal calf serum (FCS, GE Healthcare) before being deposited (300 µL) on cover slides and placed in a 24-well plate. The 24-well plate was centrifugated for 1 min at 1000 rpm at RT before the plate was incubated for 2 h at 37 °C allowing sporozoite to be deposited and glide on the slide. Thereafter the remaining medium was carefully aspirated and sporozoites were fixed with 4% PFA in PBS for 20 min at RT. After three washing steps with PBS, cells were permeabilized with 0.15% Triton X-100 in PBS for 10 min. After another three washing steps with PBS, unspecific binding was blocked by incubating cells with 3% BSA in PBS for 1 h at RT. Mouse sera diluted 1:1000 in BSA 3% in PBS and primary antibodies: (1) anti-*Pb*TRAP rabbit (kindly provided by Prof. Freddy Frischknecht, diluted 1:500 in BSA 3% in PBS); (2) anti-*Pb*CSP rabbit (Eurogentec, diluted 1:1000 in BSA 3% in PBS); (3) anti-*Pf*CSP (obtained through BEI Resources, NIAID, NIH: Monoclonal Anti-*Pf*CSP, Clone 2A10 (produced in vitro), MRA-183A, contributed by Elizabeth Nardin, diluted 1:1000 in BSA 3% in PBS) were given on the cover slides and these then incubated for 1 h at RT. After washing three times with PBS, fluorescently labeled secondary antibodies diluted in BSA 3% in PBS were added: (1) anti-rabbit Cy5 (Dianova; 1:2000); (2) anti-mouse Alexa488 (Invitrogen A-11001, 1:2000); (3) anti-mouse Alexa594 (Invitrogen A-11032 1:2000), and the samples were incubated for 1 h at RT protected from light. Washing three times with PBS was followed by a 10-min incubation with DAPI (Sigma, D9542, 1 μg/mL) for 10 min. Cover slides were then washed three times with PBS and mounted onto microscopy slides using Dako Fluorescence Mounting Medium. Stained sporozoites were imaged on a Leica TCS SP8 laser-scanning confocal microscope. Acquired images were processed with FIJI software.

In the control staining named “no serum”, no mice sera were added to sporozoites, but only secondary antibodies to control for the unspecific binding of these secondary antibodies to sporozoites.

### Statistics

Data are presented as mean ± SEM and were statistically analyzed using ordinary one-way ANOVA with Tukey correction for multiple comparisons or Log-rank (Mantel–Cox) test for survival curves. Pearson correlation coefficient was calculated to assess linear relationship between the number of targeted NANP repeats and the induction of r*Pf*CSP-specific high-avidity IgG. A saturation curve fit was applied using nonlinear regression to model induction of r*Pf*CSP-specific IgG/IgM as a function of targeted NANP repeats. Analyses were performed using GraphPad PRISM 10.3 (GraphPad Software Inc., USA). The value of *p* < 0.05 was considered statistically significant. Statistical significance is noted in figures as *p < 0.05, **p < 0.01, ***p < 0.001, ****p < 0.0001.

## Supplementary information

Supplementary information

## Data Availability

The data supporting the findings of the study are available from the corresponding author upon request.
