# Supplementary material for: Probing novel epitopes on the Plasmodium falciparum circumsporozoite protein for vaccine development
Source: NPJ Vaccines. 2024 Nov 18;9:225. doi: 10.1038/s41541-024-01006-8 (PMC11574195; doi:10.1038/s41541-024-01006-8)
Supplement: Supplementary file 1 — Supplementary information [file 41541_2024_1006_MOESM1_ESM.pdf]

Supplementary

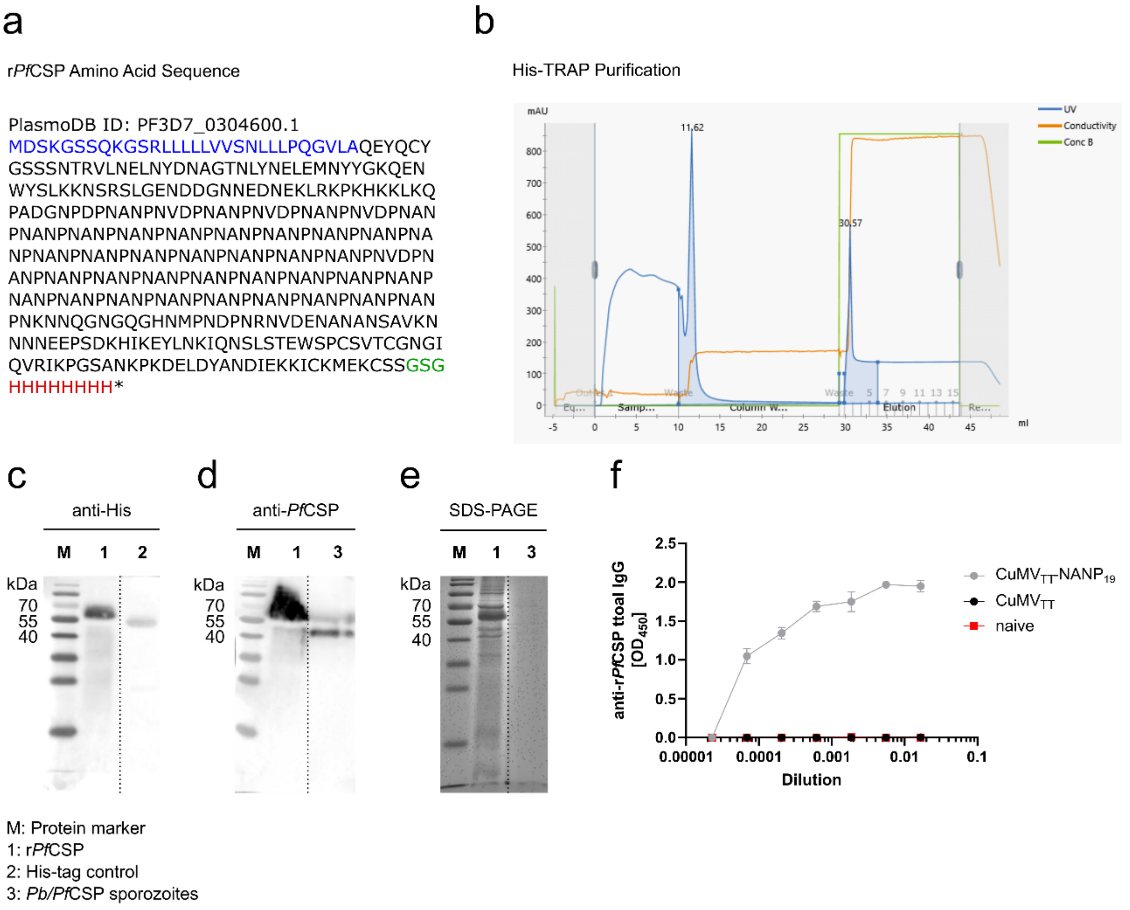

Supplementary Figure 1

Supplementary Figure 1: Characterization of recombinantly expressed r*Pf*CSP. r*Pf*CSP was expressed as previously described by *Kisalu et al.(41)*. a) Amino acid sequene of r*Pf*CSP (Pf3D7 strain; PlasmoDB ID: PF3D7_0304600.1). The first 20 leader amino acids were changed to a mammalian secretory signal peptide derived from the modified bovine lactine (blue), and the GPI-anchor residues were replaced with a GSG-linker (green) followed by a histidine-tag (red). b) Affinity chromatography (His-Trap HP column) of r*Pf*CSP after expression in Expi293™ HEK cells. c) Western blot specific for histidine. M. protein marker, 1. r*Pf*CSP, 2. control protein with histidine-tag. d) Western blot specific for *Pf*CSP. M. protein marker, 1. r*Pf*CSP, 3. *Pb*/*Pf*CSP sporozoites. e) 12 % SDS-PAGE of M. protein marker, 1. r*Pf*CSP, 3. *Pb*/*Pf*CSP sporozoites. Binding capacity of $CuMV_{TT}$-$NANP_{19}$-induced IgG antibodies to r*Pf*CSP tested by ELISA. Serum samples from $CuMV_{TT}$-immunized or naïve mice were used as controls. OD_{450} absorbance of the titration curve is shown. N = 2.

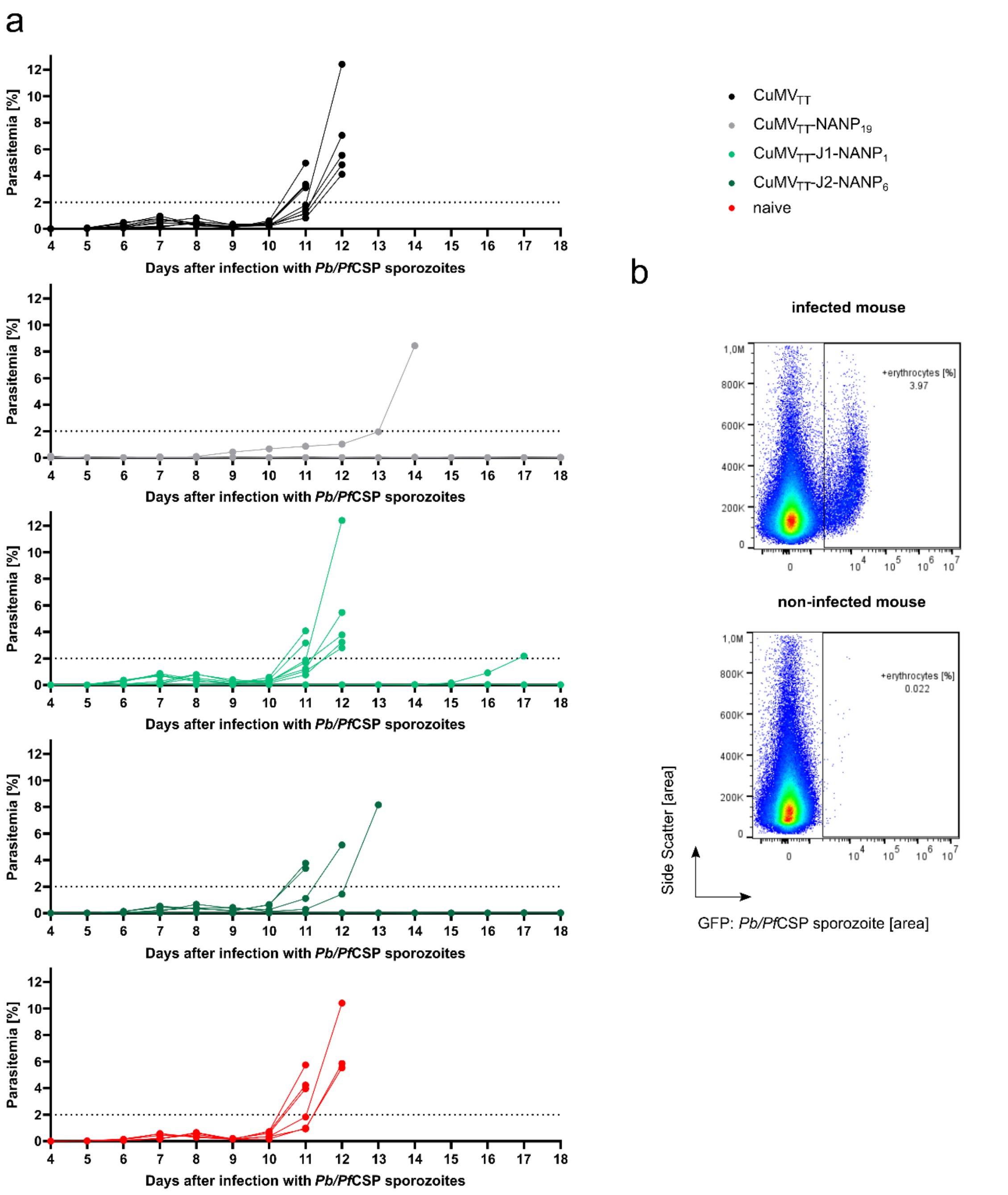

Supplementary Figure 2

Supplementary Figure 2: Parasitemia in mice infected with *Pb/Pf*CSP sporozoites following immunization with CuMV$_{TT}$-based malaria vaccine candidates (data corresponding to Figure 2f). a) Parasitemia of CuMV$_{TT}$, CuMV$_{TT}$-NANP$_{19}$, CuMV$_{TT}$-J1-NANP$_{1}$, CuMV$_{TT}$-J2-NANP$_{6}$ immunized, and naïve mice after challenge with 5000 *Pb/Pf*CSP sporozoites injected i.d. b) Parasitemia was measured by flow cytometric detection of GFP-positive (*Pb*-infected) erythrocytes. Representative flow cytometry plots of an infected and a non-infected mouse are shown. N = 9.

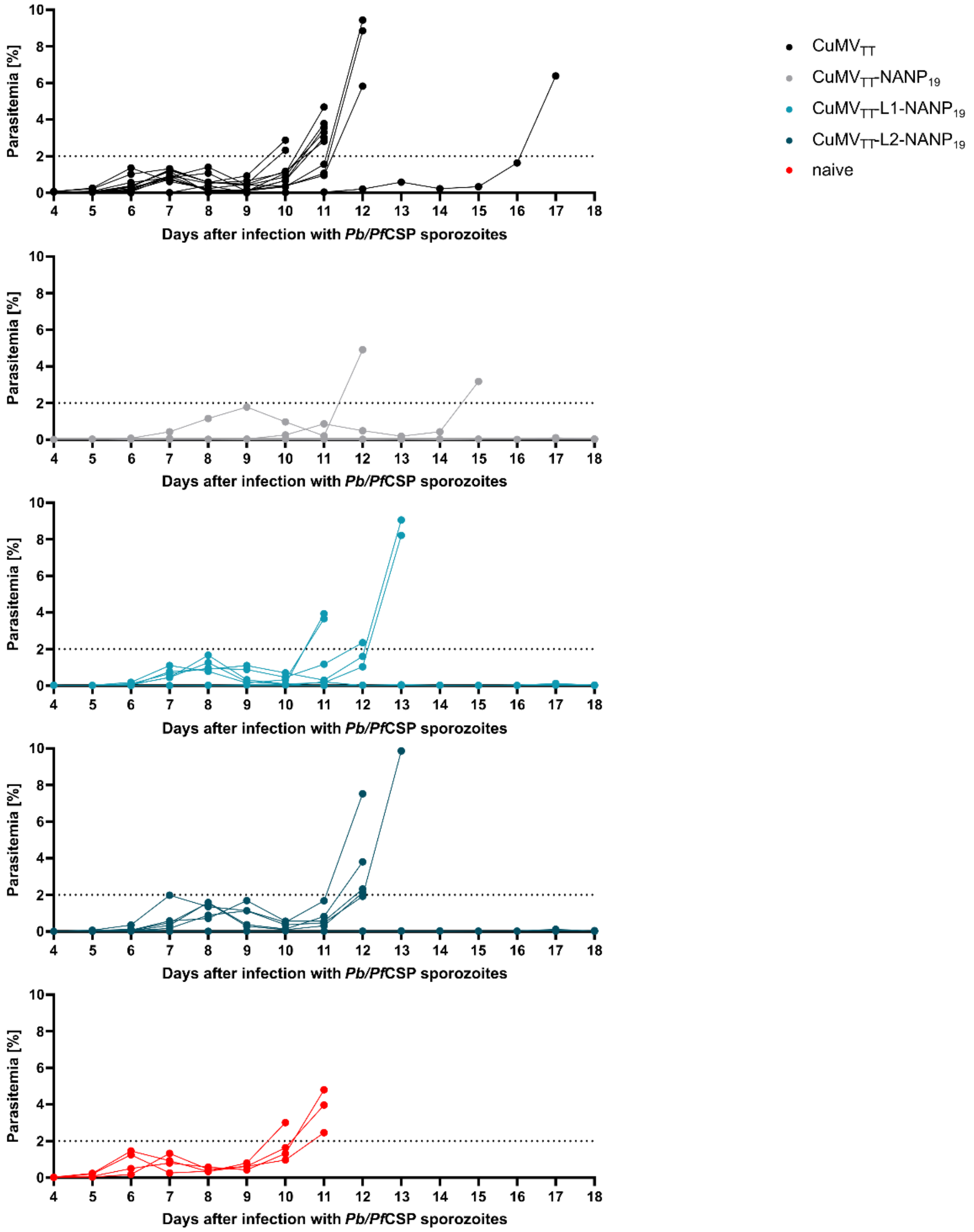

Supplementary Figure 3

Supplementary Figure 3: Parasitemia in mice infected with *Pb/Pf*CSP sporozoites following immunization with optimized $CuMV_{TT}$-based malaria vaccine candidates (data corresponding to Figure 4g). Parasitemia of $CuMV_{TT}$, $CuMV_{TT}$-$NANP_{19}$, $CuMV_{TT}$-L1-$NANP_{19}$, $CuMV_{TT}$-L2-$NANP_{19}$ immunized, and naïve mice after challenge with 5000 *Pb/Pf*CSP sporozoites injected i.d. Data of two experiments was combined. N = 12.

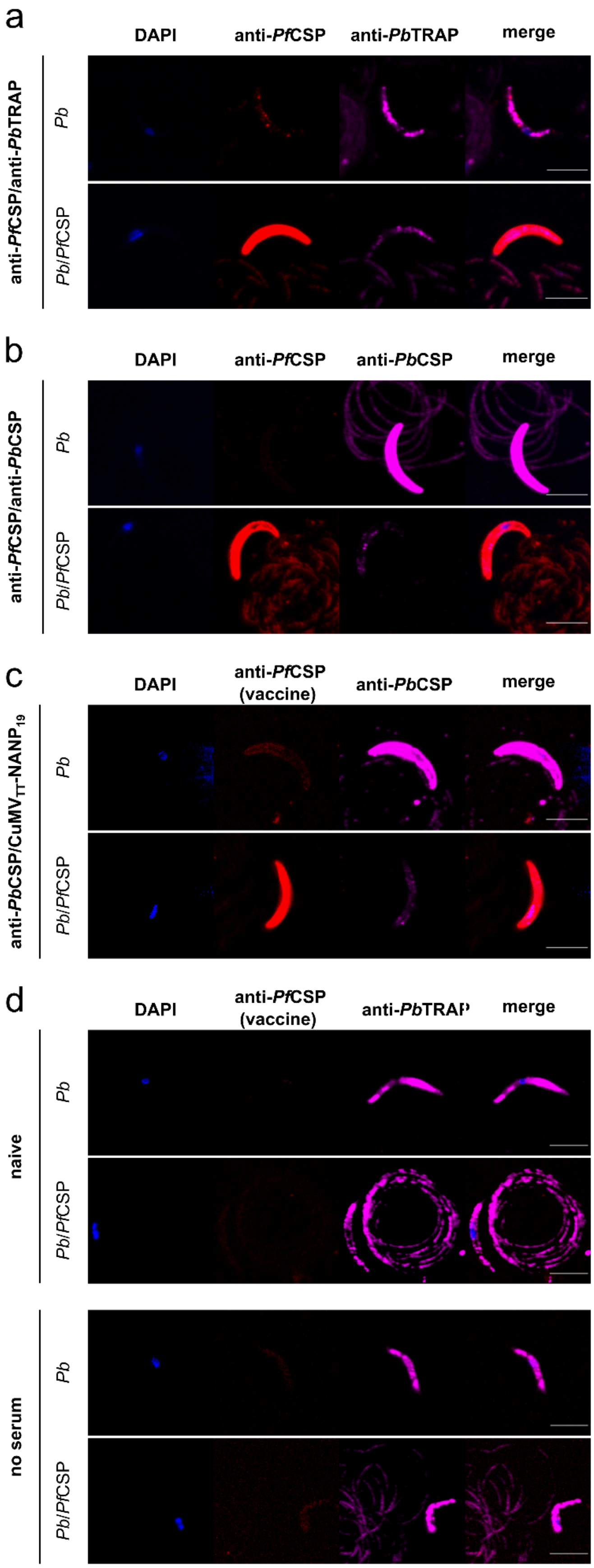

Supplementary Figure 4

Supplementary Figure 4: Binding capacity of control antibodies to *Pb/Pf*CSP sporozoites. Immunofluorescence assay testing the binding capacity to *Pb/Pf*CSP sporozoites of anti-*Pf*CSP (red) and anti-*Pb*TRAP (purple) in a), anti-*Pf*CSP (red) and anti-*Pb*CSP (purple) in b), anti-*Pf*CSP (red) and $CuMV_{TT}$-$NANP_{19}$-induced IgG (purple) in c), naive serum IgG or no serum IgG (red) and anti-*Pb*TRAP (purple) in d). DAPI nucleic acid stain is depicted in blue. As control *Pb* sporozoites were included in the analysis. Scale bar 5 µm. One representative of two similar experiments is shown.
